# Neutrophil‐based immunotherapy: A metabolic lens on mechanisms and therapeutic implications

**DOI:** 10.1002/ctm2.70780

**Published:** 2026-08-14

**Authors:** Wenchao Xu, Yu Su, Li Zhou, Jianzhou Liu, Junchao Guo

**Affiliations:** ^1^ Department of General Surgery Peking Union Medical College Hospital, Chinese Academy of Medical Sciences & Peking Union Medical College Beijing China; ^2^ Key Laboratory of Research in Pancreatic Tumor Chinese Academy of Medical Sciences Beijing China; ^3^ National Infrastructures for Translational Medicine Peking Union Medical College Hospital Beijing China; ^4^ State Key Laboratory of Complex, Severe, and Rare Diseases Peking Union Medical College Hospital, Chinese Academy of Medical Sciences & Peking Union Medical College Beijing China; ^5^ Institute of Clinical Medicine Peking Union Medical College Hospital, Chinese Academy of Medical Sciences & Peking Union Medical College Beijing China

**Keywords:** metabolic classification, metabolism remodelling, neutrophil, tumour immunotherapy

## Abstract

**Background:**

Tumor‐associated neutrophils (TANs) serve as pivotal immune modulators in the tumor microenvironment and exert profound, context‐dependent effects on tumor immunity, yet their metabolic features and functional implications remain insufficiently explored.

**Main body:**

Throughout their lifecycle, neutrophils continuously sense and adapt to shifting microenvironments, maintaining survival and functional homeostasis through metabolic reprogramming. As key hubs of the tumor metabolic network, TANs exhibit pronounced metabolic plasticity and heterogeneity. Whether active or passive, TAN metabolic reprogramming critically contributes to immune evasion via multiple mechanisms, including regulation of immune mediator expression and intercellular metabolite crosstalk. Current research focuses on strategies that induce durable TAN metabolic reprogramming while overcoming inherent limitations, aiming to establish an efficient and safe TAN‐directed immunotherapeutic framework. In this review, we systematically summarize recent advances in TAN metabolic reprogramming, emphasizing its intrinsic link to immunological phenotypes and spatiotemporal heterogeneity. We also propose a TAN metabolic classification framework (4‐Tier TAN‐MetaC) and provide a comprehensive overview of translational opportunities, bottlenecks, and actionable future directions for TAN metabolism‐targeted immunotherapies.

**Conclusion:**

Overall, TAN metabolic reprogramming represents a central driver of tumor immune evasion and a clinically actionable metabolic vulnerability for cancer immunotherapy.

**Key points:**

- Tumour‐associated neutrophil (TAN) metabolic reprogramming is tightly linked to their immunosuppressive functions.
- Spatiotemporal metabolic heterogeneity shapes TAN‐mediated tumour immunity.
- A metabolism‐based TAN stratification model unifies cross‐study frameworks.
- TAN metabolism‐targeted immunotherapy holds promise for improving patient prognosis.

## Graphical abstract

TAN metabolic reprogramming critically contributes to tumour immune evasion. A 4‑Tier TAN‑MetaC classification framework is proposed, along with an overview of translational opportunities and bottlenecks for TAN metabolism‑targeted immunotherapies.

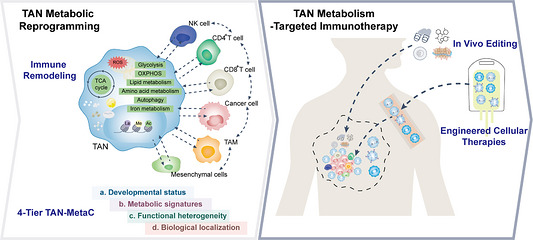

## 1 INTRODUCTION

Immunotherapies, including adoptive cell transfer (ACT), have revolutionised clinical cancer management; however, their efficacy remains limited in most solid tumours.^1^ Under physiological conditions, neutrophils are short‐lived cells whose activation and mobilisation are tightly regulated by the host to prevent their highly toxic responses from causing collateral damage to normal tissues.^2^ However, under pathological conditions, persistent neutrophil infiltration can drive chronic inflammation and tumour progression.^3^ Recent studies have revealed that tumour‐associated neutrophils (TANs) play a critical role in driving tumour immune evasion and mediating immunotherapy resistance. For instance, in the bone metastatic microenvironment, TANs promote the formation of an immunosuppressive niche through the upregulation of key immunomodulatory mediators such as chitinase‐3‐like protein 3 (CHI3L3).^4^ The expression level of unconventional myosin IF (MYO1F) in TANs has also been identified as a predictive biomarker for clinical response to immune checkpoint inhibitors.^5^ Therefore, a comprehensive understanding of the functional roles and regulatory networks of TANs in tumour immunity is essential to overcome the clinical challenge of poor immunotherapy responses.

Recent advances have established metabolic reprogramming as a fundamental determinant of TAN functional states.^6^, ^7^ As critical nodes within the complex cancer metabolic network, TANs abundantly infiltrating tumours exhibit remarkable plasticity and complex spatiotemporal metabolic heterogeneity, which collectively shape their immunomodulatory phenotypes. Technological advancements, including spatial metabolomics, have substantially deepened our understanding of key metabolic regulatory pathways, critical molecular nodes and the intricate metabolic networks that link metabolic remodelling to TAN immune functions, thereby providing a crucial theoretical foundation for the development of novel therapeutic strategies. Concurrently, the robust tissue affinity of TANs, the key role of metabolism in shaping TAN functions, and ongoing technological advances collectively make TAN‐directed metabolic interventions a promising immunotherapeutic strategy. In this review, we summarise current knowledge of neutrophil metabolism, focusing on the link between TAN metabolic reprogramming and anti‐tumour immunity, and discuss the translational opportunities, challenges and future directions of TAN‐targeted metabolic therapies.

## 2 METABOLIC LANDSCAPE OF NEUTROPHILS

### 2.1 Maturation

Neutrophil maturation is accompanied by progressive metabolic rewiring. Haematopoietic stem cells (HSCs) rely mainly on glycolysis, while granulocyte‐monocyte progenitors enhance mitochondrial respiration, reflecting a metabolic shift in early myeloid differentiation.^8^, ^9^ Autophagy‐dependent fatty acid oxidation (FAO) and oxidative phosphorylation (OXPHOS) are essential to fuel neutrophil differentiation; pharmacological or genetic ablation of metabolic rewiring blocks maturation. In contrast, excess glycolysis correlates with defective differentiation, underscoring the necessity of balanced metabolic flux for normal development.^10^ Trained immunity studies also suggest that early metabolic alterations causally influence mature cell functions. β‐glucan priming of progenitors induces sustained metabolic and epigenetic adaptations, including elevated IL‐1β/GM‐CSF‐driven glycolysis, cholesterol biosynthesis and type I interferon‐mediated mitochondrial respiration. Primed neutrophils exert stronger anti‐tumour activity, and disrupting related signalling or metabolism abolishes β‐glucan‐induced functional alterations.^11^, ^12^

### 2.2 Chemotaxis

Neutrophil chemotaxis relies heavily on metabolic reprogramming, with glycolysis serving as the primary ATP source. Increased glucose uptake supports neutrophil migration,^13^ whereas loss of the glucose‐6‐phosphate transporter greatly impairs chemotactic function.^14^ In addition, neutrophils amplify directional chemotactic signals through polarised ATP release at the cell leading edge. Secreted ATP activates front‐localised P2Y2 purinergic receptors, and its hydrolysed product adenosine acts on A3 receptors to promote forward migration. Meanwhile, A2a receptors at the cell rear trigger cAMP accumulation to drive uropod retraction, forming a set of antagonistic purinergic signals that coordinate directional movement.^15^ Upon chemotactic stimulation, mitochondria serve as a crucial ATP source for this autocrine signalling network. P2Y2 receptor activation further strengthens mTOR signalling and enhances mitochondrial function at the leading edge, including elevated calcium uptake and stabilised membrane potential. Disruption of mitochondrial ATP production via CCCP treatment or genetic ablation of key mitochondrial metabolic molecules markedly impairs neutrophil chemotaxis.^16^, ^17^ Collectively, mitochondrial metabolism, purinergic signalling and mTOR pathway activity form an integrated regulatory axis that governs gradient sensing and directional migration in neutrophils. Notably, in vitro experiments show that glutamine directly reduces neutrophil rolling velocity, adhesion and β2 integrin activation. Mechanistically, this appears to depend on transcriptomic reprogramming‐mediated alterations in mitochondrial and glutathione metabolism, though the underlying mechanisms remain to be elucidated.^18^

### 2.3 Activation

Rapid metabolic rewiring upon neutrophil activation underpins potent ROS synthesis. In vitro stimuli elicit robust oxidative burst, concurrent with reduced glycolysis and redirected glucose‐6‐phosphate toward the pentose phosphate pathway (PPP), which rapidly elevates nicotinamide adenine dinucleotide phosphate hydrogen (NADPH) availability. Blocking PPP activity substantially suppresses NADPH production and effector function, confirming the causal function of this pathway.^19^ Pyruvate kinase M2 (PKM2) activity also contributes to ROS production by modulating the availability of phosphoenolpyruvate and dihydroxyacetone phosphate (DHAP), thereby regulating NADPH oxidase activation.^20^ Under glucose limitation, neutrophils rely on alternative substrates, including fatty acids and glutamine, to maintain NADPH levels and redox balance.^9^, ^21^ Furthermore, hypoxia regulates neutrophil activation by redirecting glycolytic flux. Glycolytic intermediates are diverted to mitochondrial glycerol‐3‐phosphate dehydrogenase to sustain membrane potential and increase mitochondrial ROS (mROS) generation. This cascade stabilises hypoxia inducible factor‐1 (HIF‐1α), consequently inducing proinflammatory gene transcription and augmenting neutrophil effector responses.^22^

### 2.4 NETosis

Neutrophil extracellular trap formation (NETosis) is precisely regulated by neutrophil metabolic state. Classical suicidal NETosis is NADPH oxidase (NOX2)‐mediated and relies on PPP and glycolysis reprogramming to support ROS production. In contrast, receptor‐mediated vital NETosis can proceed via ROS‐independent pathways.^23^ In contrast, elevated glycolytic flux appears to be a shared requirement for both forms of NETosis. Preliminary evidence suggests pharmacological inhibition of hexokinase or glucose depletion markedly suppresses NETosis in response to diverse stimuli.^24^, ^25^ NETosis is also coordinately regulated by metabolism reshaping induced by diverse microenvironmental cues. Lactate accumulation elevates mROS production to facilitate NETosis, and inhibition of lactate dehydrogenase (LDH) markedly abrogates this process.^26^, ^27^ Alkaline pH and hyponatremia boost mROS generation and promote NETosis,^28^, ^29^ whereas hyperosmotic conditions suppress NOX2‐dependent NETosis by reducing ROS synthesis.^30^ In addition, one study reported that tumour‐derived nicotinamide phosphoribosyltransferase (NAMPT) can also activate sirtuin 1 (SIRT1), trigger neutrophil polarisation and mitochondria‐dependent NETosis, and ultimately accelerate breast cancer lung metastasis.^31^ However, the link between nicotinamide metabolism and neutrophil function requires further exploration.

### 2.5 Degranulation

Neutrophils utilise glycolysis to fuel degranulation. A key supporting study demonstrated that 2‐deoxy‐D‐glucose (2‐DG) treatment markedly inhibits granule release.^32^ The glycolytic enzyme PKM2 further exerts a non‐metabolic regulatory role by directly phosphorylating synaptosome‐associated protein 23 (SNAP‐23), thereby accelerating granule–plasma membrane fusion.^33^ Mitochondrial function has also been found to play a role in degranulation, although the underlying mechanisms remain incompletely understood. One study reported that neutrophils with dysfunctional NOX2 exhibit enhanced degranulation compared to wild‐type cells, suggesting a negative regulatory role of NOX2‐derived ROS in this process.^34^ Pharmacological evidence suggests that mitochondria‐targeted antioxidants suppress the release of primary and secondary granules by reducing mROS generation.^35^

## 3 TAN METABOLIC REMODELLING AND ANTI‐TUMOUR IMMUNITY

### 3.1 Glucose metabolism

**FIGURE 1:**
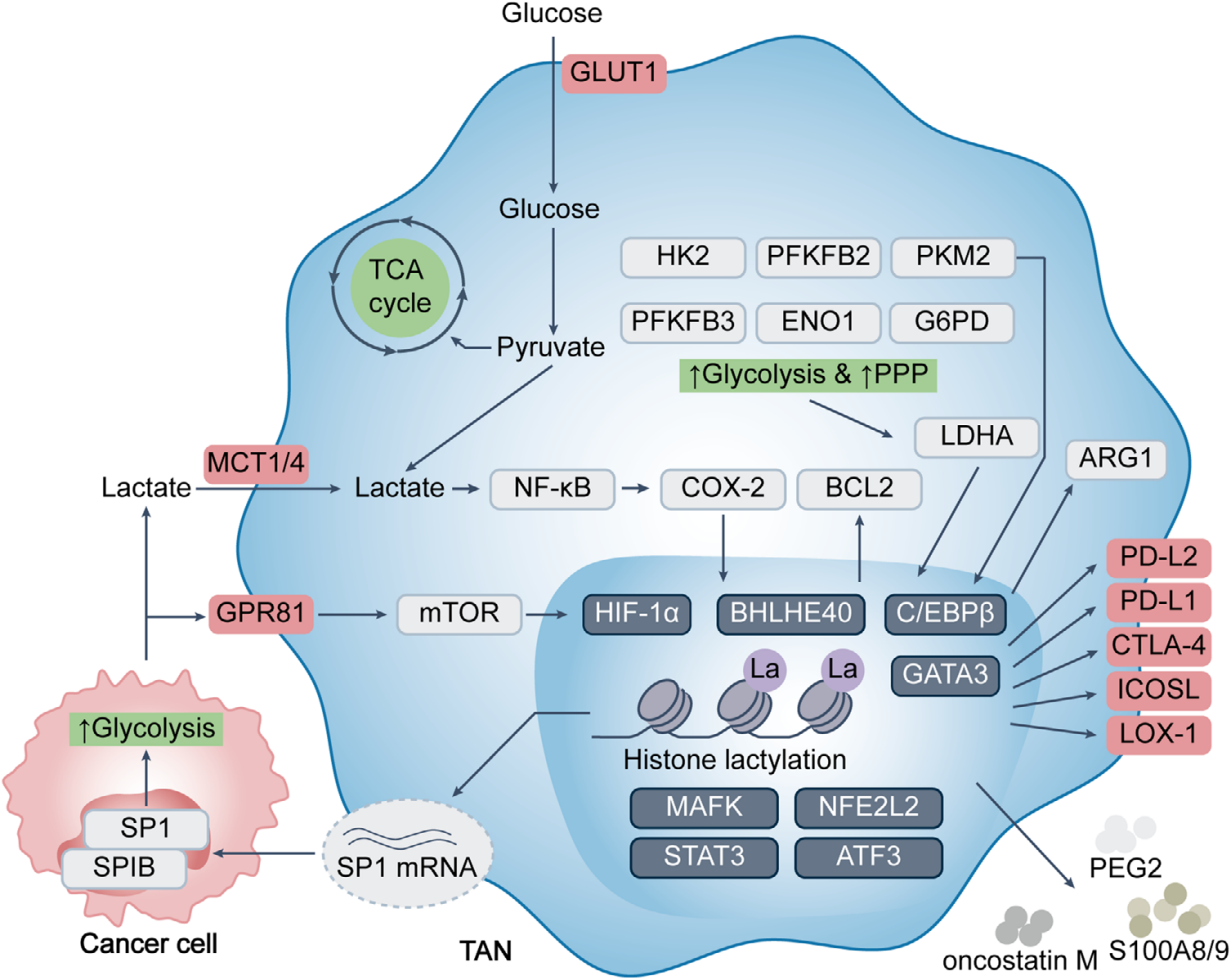
Immunometabolic regulation on TAN glucose metabolism. In the glucose‐depleted, lactate‐rich tumour microenvironment (TME), infiltrated tumour‐associated neutrophils (TANs) elevate glucose transporter 1 (GLUT1) and core enzymes of glycolysis and the pentose phosphate pathway (PPP) to accelerate glycolytic metabolism. Extracellular lactate is imported via monocarboxylate transporters 1/4 (MCT1/4) and recognised by G protein‐coupled receptor 81 (GPR81) to initiate mechanistic target of rapamycin (mTOR) signalling and histone lactylation. Activated transcription factors downstream of these metabolic events induce the transcription of various immune checkpoints and immunosuppressive effector molecules. In parallel, TAN‐secreted *Salmonella* pathogenicity island 1 (SP1) mRNA is transmitted to tumour cells to further boost tumoural glycolysis and lactate generation, forming a sustained positive feedback metabolic loop. Such metabolic rewiring empowers TANs to effectively suppress cytotoxic T‐cell responses and promote tumour immune escape.

TANs sustain survival and immunomodulatory functions via adaptive glucose reprogramming, primarily relying on anaerobic glycolysis and PPP (Figure 1). Multiple independent studies have indicated that the hypoxic, glucose‐deprived and lactate‐rich tumour microenvironment (TME) elicits diverse cellular stresses in TANs. These stresses upregulate stress‐responsive transcription factors including HIF‐1α and basic helix‐loop‐helix family member E40 (BHLHE40) to initiate transcription of glycolytic genes and remodel epigenetic landscapes to open chromatin regions surrounding glycolytic loci.^36^, ^37^, ^38^ TANs in lung cancer tissues display increased glucose transporter 1 (GLUT1) expression and glycolytic activity; specific ablation of *Glut1* in TANs attenuates their pro‑tumourigenic and radioresistance‑promoting functions.^39^ Consistently, TANs derived from hepatocellular carcinoma tissues exhibit upregulated expression and enzymatic activity of glycolytic enzymes as well as the PPP rate‑limiting enzyme glucose‐6‐phosphate dehydrogenase, which mediate ROS‐dependent NETosis and facilitate tumour progression.^40^ In addition, enhanced glycolysis activates the lactate dehydrogenase A (LDHA)‐signal transducer and activator of transcription 3 (STAT3) axis to upregulate programmed cell death ligand 1 (PD‐L1) expression and promote oncostatin M secretion, thereby blunting cytotoxic T‐cell anti‐tumour responses.^41^ Treatment with retinoic acid or 2‐DG suppresses glycolytic activity, which subsequently triggers activation of the AMPK pathway and ultimately represses CCAAT/enhancer binding protein beta (C/EBPβ)‐ and arginase 1 (ARG1)‐mediated TAN immunosuppression.^42^ The PKM2 modulates GATA binding protein 3 (GATA3) activity to bidirectionally regulate PD‐L1 and PD‐L2; however, PKM2 inhibition uniformly downregulates both checkpoints in mouse xenograft models. These findings indicate a species‐dependent PKM2–GATA3 regulatory axis, suggesting PKM2 as a promising immunotherapeutic target.^43^

HIF‐1α is an important immunoregulatory molecule in TAN function. HIF‐1α shows higher expression levels than circulating and bone marrow neutrophils and directly activates transcription of glycolytic genes. Enhanced glycolysis further stabilises HIF‐1α through glycerol 3‐phosphate shuttle‐dependent ROS generation, establishing a glycolysis–HIF‐1α positive feedback loop.^22^, ^44^ Under hypoxia, Hif‐1α directly drives *Cd274 (encoding Pd‐l1)* transcription of TANs to suppresses T‐cell cytotoxicity in multiple tumour models,^45^ and neutrophil‐specific *Hif1a* deletion reinstates NK and CD8^+^ T‐cell activation, reduces tumour burden, and improves host survival.^46^

As a critical glycolytic end product, lactate is considered to play an important role in regulating TAN immune functions. Independent mechanistic studies indicate that lactate can elevate the expression of anti‐apoptotic and immunosuppressive genes in TANs by increasing histone lactylation levels, and glycolysis inhibition by 2‐DG reverses these modifications.^47^, ^48^ Lactate also drives TAN polarisation and upregulates immune checkpoint molecules through the GPR81/mTOR/HIF‐1α/STAT3 and MCT1/NF‐κB/COX‐2 signalling cascades.^49^, ^50^ In addition, one study has reported that polarised TANs release *Salmonella* pathogenicity island 1 (SP1) mRNA‐containing extracellular vesicles to upregulate SP1 expression in tumour cells; SP1 cooperates with an SPI‐1‐related protein to further enhance tumour glycolysis, glucose uptake and lactate secretion, thereby positively reinforcing lactate‐mediated TAN immunosuppression.^51^

Although enhanced glycolysis is commonly associated with TAN immunosuppression, conflicting evidence has also been reported. For instance, in pancreatic cancer liver metastases, P2RX1^−^ immunosuppressive TAN subsets exhibit elevated inhibitory molecular profiles, but possess diminished glycolytic activity and enhanced OXPHOS capacity.^52^ Single‐cell transcriptomic analyses of clinical pancreatic liver metastasis samples also suggest that pro‐tumour TAN subsets do not rely on robust glycolytic activation. Distinct TAN subsets show no single dominant metabolic pathway, with neither pro‐tumour nor anti‐tumour subsets displaying universally biased metabolic signatures.^7^ Beyond the inherent methodological differences between functional metabolic assays and single‑cell transcriptomics, several additional factors contribute to the observed heterogeneity in the glucose metabolism–TAN immunophenotype association, including the lack of standardised TAN classification criteria, variations in tumour type (e.g., hepatocellular carcinoma vs. pancreatic cancer), and the spatiotemporal heterogeneity of TANs (e.g., primary vs. metastatic lesions; bulk tissue analysis vs. spatially resolved multi‑omics profiling). Based on these observations, it can be inferred that glycolysis and OXPHOS may serve as mutually compensatory rather than mutually exclusive metabolic programmes depending on the context. Pharmacological evidence suggests that glycolysis inhibition can amplify OXPHOS‐dominant TAN immunosuppression.^9^ However, this inference remains to be quantitatively validated, for instance, through isotope‐tracing metabolic flux analysis to determine the relative substrate utilisation and pathway engagement under distinct nutrient conditions.

### 3.2 Mitochondrial metabolism

**FIGURE 2:**
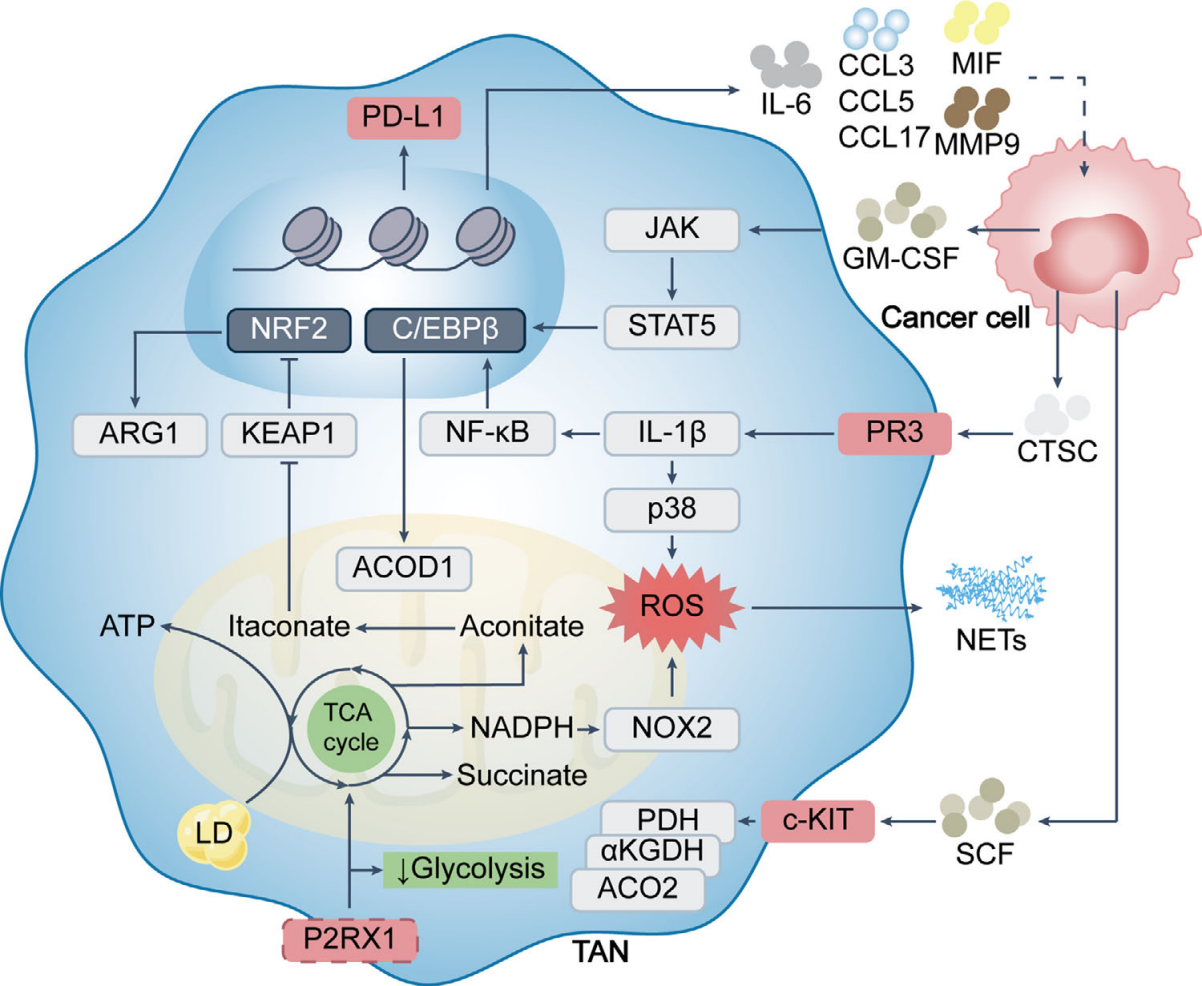
Immunometabolic regulation by TAN mitochondrial metabolism. Within the tumour microenvironment (TME), tumour‐derived granulocyte‐macrophage colony‐stimulating factor (GM‐CSF), stem cell factor (SCF) and cathepsin C (CTSC) induce mitochondrial metabolic reprogramming in TANs, featuring enhanced tricarboxylic acid (TCA) cycling, succinate buildup and aconitate decarboxylase 1 (ACOD1)‐dependent itaconate generation. Itaconate regulates Kelch‐like ECH‐associated protein 1 (KEAP1)/nuclear factor erythroid 2‐related factor 2 (NRF2) signalling to influence arginase 1 (ARG1) and programmed death‐ligand 1 (PD‐L1) expression, while NADPH oxidase 2 (NOX2)‐mediated reactive oxygen species (ROS) accumulation promotes neutrophil extracellular trap formation (NETosis). The CTSC‐proteinase 3 (PR3)‐interleukin‐1β (IL‐1β) axis amplifies inflammatory signalling via p38 mitogen‐activated protein kinase (p38) and nuclear factor kappa‐B (NF‐κB), and the GM‐CSF‐triggered Janus kinase‐signal transducer and activator of transcription 5‐CCAAT/enhancer‐binding protein β (JAK‐STAT5‐C/EBPβ) cascade further consolidates immunosuppressive transcriptional programmes. TANs subsequently secrete abundant cytokines, chemokines and proteases to reciprocally facilitate immunosuppression.

To address the mechanistic basis of this inferred metabolic flexibility, this section examines how mitochondrial metabolism fine‐tunes TAN immune phenotypes through redox signalling and tricarboxylic acid (TCA) cycle‐dependent regulation (Figure 2). mROS serves as a key redox signalling hub. Functional validation has confirmed that pharmacological ROS scavenging effectively abrogates TAN‐driven T‐cell inhibition,^52^ suggesting that mROS plays an important role in TAN‐mediated immunosuppression. Inflammatory cues within the TME, including GM‐CSF, LPS and IFN‐γ, can trigger robust mROS accumulation, which initiates nuclear factor‐erythroid 2‐related factor 2 (Nrf2)‐dependent antioxidant responses and transcriptionally upregulates the key immunosuppressive effectors PD‐L1 and ARG1.^52^ Preliminary evidence suggests that tumour cells sustain persistent mROS production in TANs via aberrant SCF/c‐Kit signalling, which preserves mitochondrial function and intracellular NADPH homeostasis to stabilise chronic redox stress.^9^ Consistently, tumour‐derived cathepsin C potentiates mROS buildup in TANs within breast cancer metastatic lesions by activating the PR3/IL‐1β cascade, thereby facilitating excessive NETosis and further remodelling a pro‐tumour TME.^53^ In addition, under metabolic stress in hepatic metastatic niches, immature low‐density neutrophils (iLDNs) adopt lipid droplet‐dependent mitochondrial metabolism to sustain ATP replenishment and promote NETosis, which greatly supports metastatic progression.^54^

TCA cycle metabolites are also considered to participate in regulating TAN survival and immune function. One study has found that tumour‑secreted GM‑CSF activates the STAT5–C/EBPβ axis to induce aconitate decarboxylase 1 (ACOD1) expression, which catalyses the conversion of citrate to itaconate. Itaconate activates Nrf2 antioxidant signalling to resolve excessive ROS‑driven ferroptosis, thereby prolonging TAN intratumoural survival and maintaining stable immunosuppressive programmes.^55^ As a key TCA intermediate, succinate acts as a versatile metabolism‑immune mediator in immune cells. Previous studies have found that succinate can promote neutrophil activation, tissue infiltration and NETosis, but the potential role in remodelling TAN immune phenotypes remains unexplored.^56^, ^57^, ^58^

Neutrophils gradually lose mitochondrial content during bone marrow maturation, and resting circulating neutrophils exhibit minimal basal mitochondrial abundance and activity.^59^ In the tumour context, dormant mitochondrial metabolism in TANs can be potently reactivated and participate in tumour immune modulation. Furthermore, as a core regulator of TAN survival, targeting mitochondria and their downstream signalling may represent a promising translational strategy to extend TAN lifespan and reshape their effector functions.^60^

### 3.3 Lipid metabolism

Under physiological conditions, resting neutrophils contain minimal lipid droplets and exhibit low basal lipid metabolic activity. In contrast, microenvironmental stimulation induces robust lipid metabolic reprogramming in TANs, which profoundly remodels their functional properties.^61^ In multiple tumour models, tumour‐derived GM‐CSF/G‐CSF activate STAT3/STAT5 signalling to transcriptionally upregulate key lipid transporters, including cluster of differentiation 36 (CD36), fatty acid transport proteins (FATPs), and very low‐density lipoprotein receptor (VLDLR), thereby potentiating lipid uptake.^62^, ^63^ FATP2 acts as a TAN‐specific, indispensable immunomodulatory lipid transporter, and the excessive lipid accumulation mediated by FATP2 enhances ROS production. Genetic evidence suggests that *Slc27a2 (encoding Fatp2)* ablation completely abolishes the immunosuppressive activity of TANs,^61^ while pharmacological studies have confirmed that FATP2 inhibition downregulates immunosuppressive molecules including ARG1, inducible nitric oxide synthase (iNOS), and S100 calcium‐binding protein A9 (S100A9), reduces ROS‑mediated TAN immunosuppression, and restores T‐cell effector functions.^64^ Although FATP2 is not an exclusive transporter for arachidonic acid (AA), its overexpression markedly promotes AA uptake and intracellular accumulation in TANs. One study reported that elevated AA levels are closely correlated with increased PD‐L1 expression and immunotherapy resistance.^65^ Mechanistic studies indicate that excess AA is further converted to prostaglandin E2 (PGE_2_) via cyclooxygenase‐2 (COX2) catalysis, triggering two distinct immunosuppressive cascades. On one hand, PGE_2_ suppresses RIPK3 signalling through the PKA/CREB axis and establishes an NF‐κB/COX2‐positive feedback loop to sustain autocrine PGE_2_ production and ARG1 upregulation.^66^ On the other hand, PGE_2_ binds E‐type prostanoid 2/4 (EP2/EP4) receptors to block STAT1 phosphorylation and abrogate IFN‐γ‐dependent signalling, thereby preventing immunotherapy‐induced anti‐tumour TAN polarisation.^67^ AA also serves as a critical precursor for the production of oxylipins via lipoxygenase‐mediated catalysis. Oxylipin metabolites such as 15(S)‐hydroxyeicosatetraenoic acid [15(S)‐HETE] activate peroxisome proliferator‐activated receptor gamma (PPARγ), which transcriptionally regulates core inhibitory genes, including *Cd274*, *Cxcl2* and *Arg1* in myeloid cells.^68^, ^69^ In ovarian cancer, 15(S)‐HETE‐triggered PPARγ has also been found to cooperate with C/EBPβ in reshaping myeloid immunophenotypes.^70^ Collectively, these findings indicated a conserved ‘metabolite‐signalling‐transcription’ regulatory axis in myeloid cells; however, direct mechanistic validation specifically in TANs remains limited and requires further systematic investigation.

Lectin‐type oxidised LDL receptor 1 (LOX‐1) also serves as a critical lipid uptake receptor and a specific surface marker for immunosuppressive TAN subsets. LOX‐1 is barely detectable in circulating neutrophils from healthy individuals but highly expressed in nearly half of intratumoural TANs, correlating with elevated ARG1 expression and robust ROS generation.^71^, ^72^ Preliminary in vitro evidence suggests that elevated oxidised low‐density lipoprotein (oxLDL) and its derivatives (oxidised phosphatidylcholines, lysophosphatidylcholine) facilitate TAN NETosis,^73^ whereas in vivo causal evidence linking oxidised lipid metabolism to TAN‐mediated immunosuppression remains lacking.

Beyond lipid uptake, TANs undergo extensive fatty acid remodelling characterised by enhanced FAO and polyunsaturated fatty acid (PUFA) reprogramming. Preliminary studies suggest that compared with splenic and bone marrow neutrophils, TANs upregulate multiple FAO enzymes, accompanied by increased fatty acid uptake and oxygen consumption. Pharmacological studies have confirmed that FAO blockade reduces TAN‐derived ARG1, ROS, NO and peroxynitrite production and alleviates TAN‐mediated inhibition of T‐cell proliferation.^62^ In sorafenib‐treated tumour models, sorafenib activates PPARα signalling to induce FAO‐related enzymes; genetic or pharmacological inhibition of PPARα/FAO efficiently abolishes sorafenib‐elicited TAN immunosuppression.^74^ Another single‑cell transcriptomic study of cervical cancer reported that disrupted lipid metabolism as a hallmark of pro‐tumour TANs, in which elevated elongation of very long‐chain fatty acids, elongase 5 (ELOVL5) activity drives the synthesis of long‐chain PUFAs to support TAN survival and pro‐tumour function.^75^ In breast cancer, ferroptotic CD4^+^ T cells secrete CXCL3, IL‐8 and S100A9 to recruit TANs and activate IL‐1β/NF‐κB signalling, which subsequently upregulates the membrane bound O‐acyltransferase domain containing 1 (MOAT1). MOAT1 rewires TAN fatty acid preference toward PUFAs, increases TAN ferroptosis susceptibility, and promotes the expression and secretion of indoleamine 2,3‐dioxygenase (IDO), PGE_2_ and oxidised lipids to suppress CD8^+^ T‐cell proliferation and cytotoxicity.^76^

Lipid droplet homeostasis, in addition to serving as a nutrient buffer to sustain TAN survival, is also considered an important prerequisite for TAN pro‑tumour progression and immunosuppressive effects. One study has reported that lung carcinoma cell inhibits lipolysis and stabilise intracellular lipid droplets in TANs by repressing the lipolytic enzyme adipose triglyceride lipase while upregulating hypoxia‐inducible lipid droplet‐associated protein (HILPDA) and G0/G1 switch protein 2 (G0S2) expression.^77^ Microenvironmental insulin‐like growth factor‐1 (IGF‐1) and mesenchyme‐derived PGE_2_ further enhance HILPDA expression via HIF‐1α signalling to block lipid droplet degradation^78^, ^79^; consequently, lipid accumulation drives ROS generation and immunosuppressive mediator production. In colorectal cancer liver metastases, hepatic stellate cell‐derived IL‐33 activates the mTOR‐PPARγ cascade in TANs, which transcriptionally induces diacylglycerol O‐acyltransferase 1/2 (DGAT1/2) to accelerate de novo lipid droplet biogenesis. Pharmacological evidence suggests that mTOR/PPARγ inhibition effectively downregulates PD‐L1 and other immunosuppressive markers and restores CD8^+^ T‐cell proliferation, indicating an important role of the mTOR/PPARγ/lipid droplet axis in sustaining TAN immunosuppression.^80^

**FIGURE 3:**
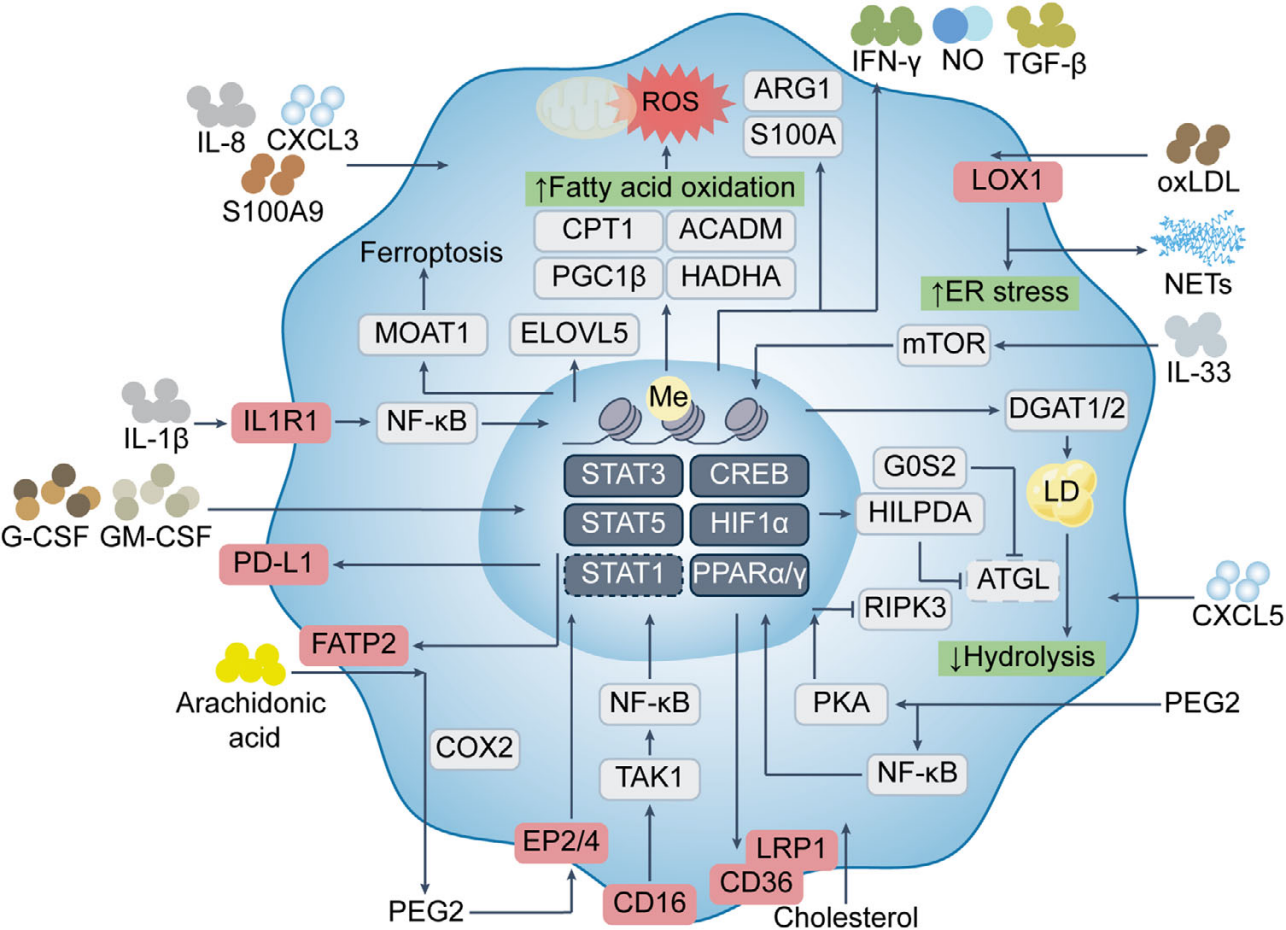
Immunometabolic regulation by TAN lipid metabolism. Tumour‐derived cytokines, lipids and chemokines trigger lipid metabolic remodelling within TANs: TANs enhance lipid uptake through fatty acid transport protein 2 (FATP2), cluster of differentiation 36 (CD36) and lectin‐like oxidised low‐density lipoprotein receptor‐1 (LOX‐1), boost fatty acid oxidation (FAO) governed by carnitine palmitoyltransferase 1 (CPT1), hydroxyacyl‐CoA dehydrogenase trifunctional multienzyme complex subunit alpha (HADHA) and related enzymes, and stabilise intracellular lipid droplets by inhibiting lipolytic breakdown. Downstream peroxisome proliferator‐activated receptor α/γ (PPARα/γ), signal transducer and activator of transcription 1/3/5 (STAT1/3/5), cAMP response element‐binding protein (CREB), hypoxia‐inducible factor 1α (HIF‐1α) and nuclear factor kappa‐B (NF‐κB) transcriptional programmes coordinate arachidonic acid‐prostaglandin E_2_ (AA‐PGE_2_) signalling, endoplasmic reticulum (ER) stress and mitochondrial reactive oxygen species (ROS) generation. Such lipid adaptations drive diverse biological outcomes: accumulation of immunosuppressive effectors, induction of ferroptosis and neutrophil extracellular trap (NETosis) formation, as well as robust secretion of interleukin‐8 (IL‐8), C–X–C motif chemokine ligand 3 (CXCL3), S100 calcium‐binding protein A9 (S100A9) and other inflammatory mediators. All these lipid‐dependent alterations endow TANs with potent capacity to suppress CD8^+^ T‐cell anti‐tumour immunity and maintain a protumourigenic immune microenvironment.

In summary, TANs exhibit active lipid metabolic remodelling (Figure 3). Although distinct TAN subsets have been found to adopt divergent lipid metabolic programmes, they ultimately appear to converge on a unified immunosuppressive state, suggesting a pattern of ‘metabolic heterogeneity with functional uniformity’. Although the correlations between lipid reprogramming and TAN immunosuppression have been well documented, systematic causal validation at the molecular level remains lacking. Future studies utilising TAN‐specific conditional knockout models and multi‐omics integration will help delineate this sophisticated regulatory network and accelerate the clinical translation of TAN‐targeted lipid metabolic immunotherapy.

### 3.4 Amino acid metabolism

**FIGURE 4:**
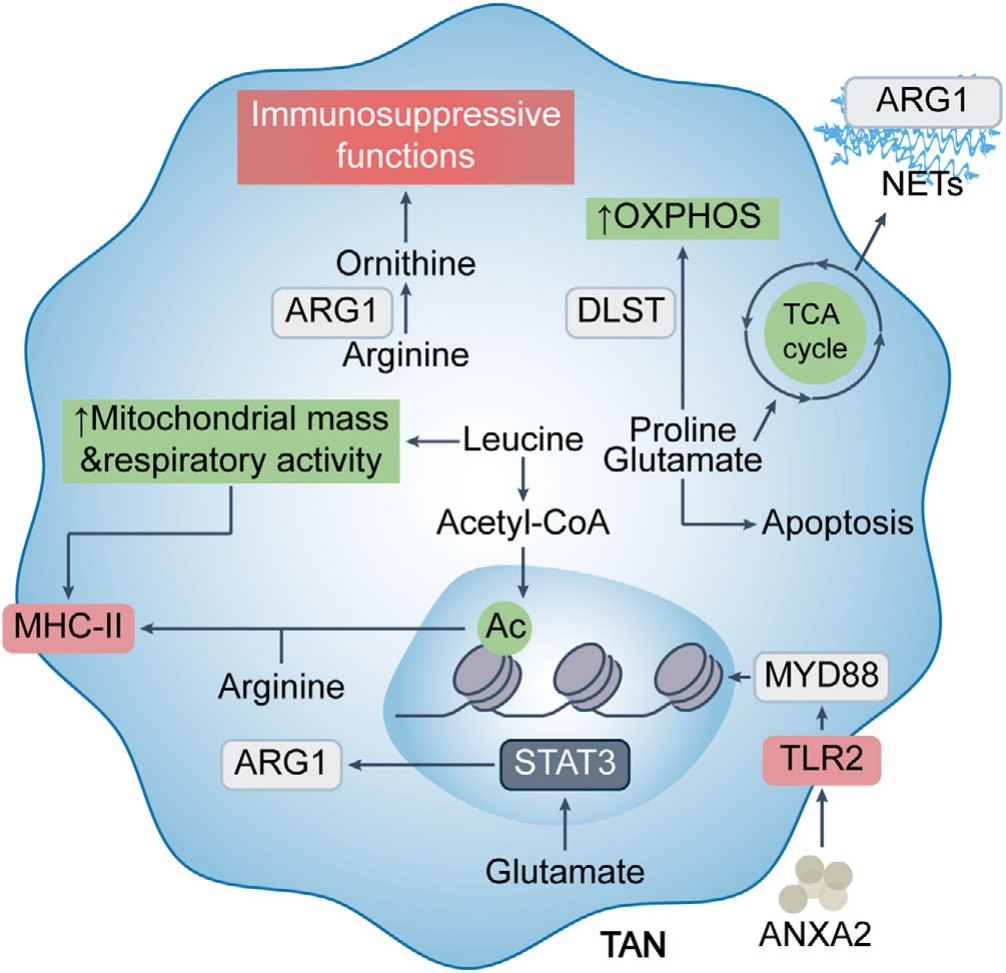
Immunometabolic regulation by TAN amino acid metabolism. Multiple amino acids exert divergent metabolic effects: glutamine and glutamate feed the mitochondrial tricarboxylic acid (TCA) cycle; leucine boosts mitochondrial mass, respiratory activity and histone acetylation to upregulate major histocompatibility complex class II (MHC‐II) for antigen presentation; proline and glutamate drive oxidative phosphorylation (OXPHOS) while simultaneously modulating apoptotic susceptibility. Meanwhile, arginase 1 (ARG1) catalyses arginine conversion to ornithine, depleting intratumoural arginine to establish robust immunosuppression. Distinct amino acid preference therefore dictates the heterogeneous functional states of tumour‐associated neutrophils (TANs) within the tumour microenvironment (TME).

Distinct amino acid utilisation preferences drive TANs toward either proinflammatory or immunosuppressive phenotypes, constituting an important factor in TAN heterogeneity (Figure 4). One cross‑cancer single‑cell profiling study demonstrated that antigen‑presenting HLA‐DR^+^ TANs are enriched in branched‐chain amino acid metabolism, whereas immunosuppressive VEGFA^+^SPP1^+^ TANs prioritise vitamin and glycosaminoglycan metabolism. Mechanistically, leucine accumulation is reported to elevate intracellular acetyl‐CoA levels. This promotes H3K27ac modification, which enhances MHC‐II gene transcription in TANs, ultimately sustaining anti‐tumour activity.^81^ When glucose supply is insufficient, neutrophils can switch to glutamine/glutamate‑dependent energy metabolism to exert immunosuppressive effects. One study has demonstrated that in the breast cancer liver metastatic niche, this metabolic adaptation sustains TCA cycle activity, promotes NETosis and establishes an immunosuppressive TME.^54^ In ovarian cancer, pharmacological studies have confirmed that TANs exhibit enhanced glutamine‑driven OXPHOS with marked dihydrolipoamide S‐succinyltransferase (DLST) upregulation; DLST inhibition reduces OXPHOS and immunosuppressive markers, improving immunotherapy efficacy.^82^ Tumour‐derived glutamate further induces STAT3 expression in TANs, which upregulates *ARG1* transcription.^83^ Consistently, the glutamate release inhibitor riluzole attenuates tumour‐secreted glutamate and reverses STAT3‐mediated TAN immunosuppressive polarisation.^84^

Arginine metabolism also shapes TAN immune function. A single‑cell sequencing study in hepatocellular carcinoma reported that TANs with low arginine metabolism exhibit poorly differentiated pro‐tumour features, whereas those with high arginine metabolism are predominantly distributed in normal tissues and display proinflammatory properties.^85^ Exogenous arginine supplementation modestly enhances HLA‐DR‐mediated TAN antigen presentation, partially restoring their immune‐activating capacity.^81^ ARG1 serves as a key enzyme in arginine catabolism and a critical molecular mediator of TAN immunosuppressive function. In non‐small cell lung cancer, the paracrine factor annexin A2 (ANXA2) induces ARG1 expression in TANs via the toll‐like receptor 2 (TLR2)/myeloid differentiation primary response 88 (MYD88) signalling cascade, whereas ANXA2‐blocking peptides effectively abrogate tumour lysate‐induced ARG1 upregulation.^86^ It has been established that immune cell activation is highly dependent on arginine supply,^87^ and preliminary evidence suggests that excessive Arg1 released by TANs depletes intratumoural arginine, thereby impairing anti‑tumour immunity.^88^

Collectively, the functional fate of TANs is highly dependent on the activation status of specific amino acid metabolic pathways. It should be noted, however, that most findings are confined to single pathways, and the integrative crosstalk among different amino acid axes remains poorly understood. Furthermore, how amino acid flux integrates intra‑ and extracellular signals and whether metabolic intermediates directly affect epigenetic regulation require further investigation using genetic and metabolic intervention approaches.

### 3.5 Autophagy and iron metabolism

**FIGURE 5:**
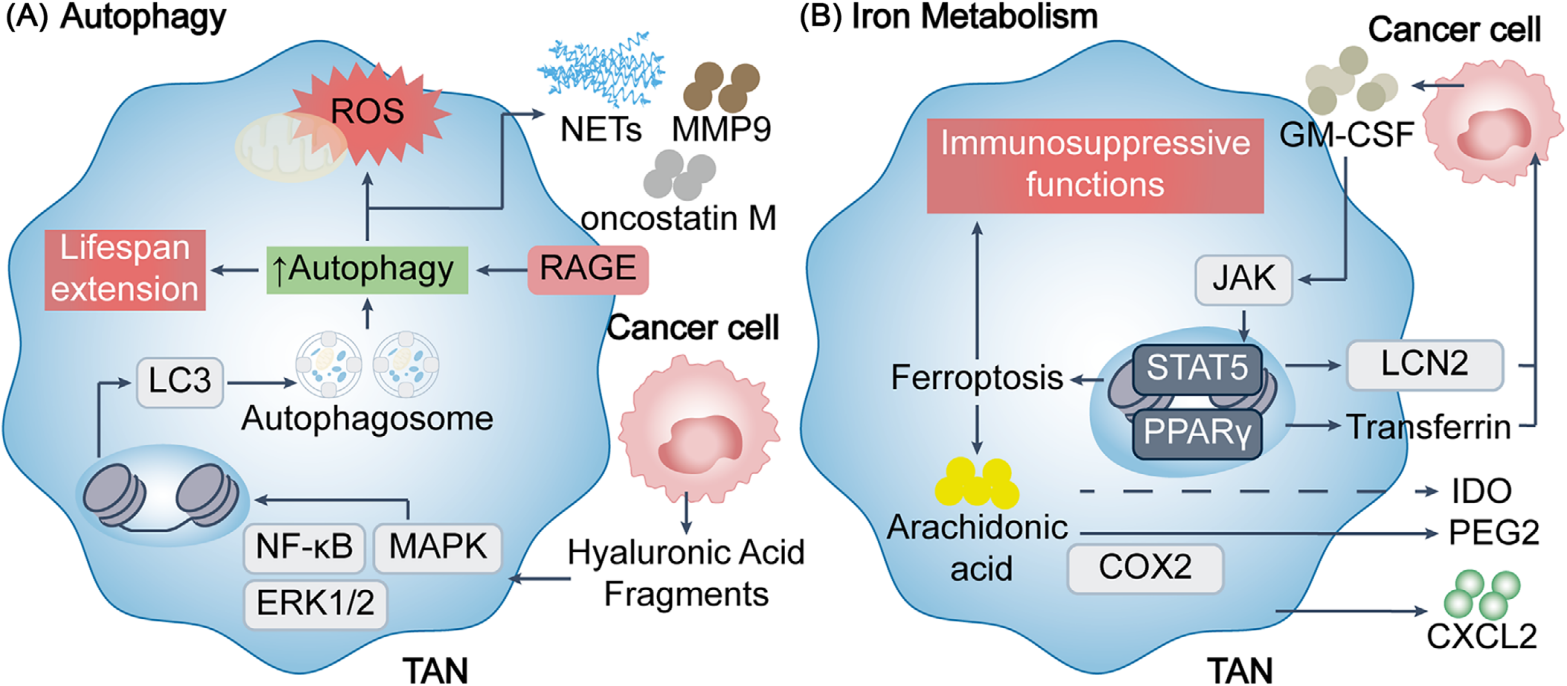
Immunometabolic regulation by TAN autophagy and iron metabolism. (A) Autophagy. Tumour‐derived hyaluronic acid fragments activate nuclear factor kappa‐B (NF‐κB)/mitogen‐activated protein kinase (MAPK)/extracellular signal‐regulated kinase 1/2 (ERK1/2) pathways to elevate autophagy, which prolongs TAN survival, induces reactive oxygen species (ROS) generation, promotes neutrophil extracellular trap (NETosis) formation, and triggers matrix metalloproteinase 9 (MMP9) and oncostatin M release. (B) Iron metabolism. Tumour‐released granulocyte‐macrophage colony‐stimulating factor (GM‐CSF) activates Janus kinase‐signal transducer and activator of transcription 5‐peroxisome proliferator‐activated receptor γ (JAK‐STAT5‐PPARγ) signalling to induce transferrin and lipocalin 2 (LCN2) expression, reshaping tumoural iron pools to fuel tumour growth. Perturbed iron metabolism sensitises TANs to arachidonic acid (AA)‐mediated ferroptosis, whereas surviving ferroptotic TANs secrete prostaglandin E_2_ (PGE_2_), indoleamine 2,3‐dioxygenase (IDO) and C–X–C motif chemokine ligand 2 (CXCL2) in a cyclooxygenase‐2 (COX2)‐dependent manner to mediate potent immune suppression and facilitate tumour immune escape.

Active autophagy has been detected in intratumoural TANs. One study has found that autophagy sustains mitochondrial homeostasis, upregulates Mcl‐1 and reduces cleaved caspase‐3 to prolong TAN survival, while promoting MMP9 and oncostatin M secretion to impair anti‐tumour surveillance and form an immunosuppressive TME.^89^ Another study found that autophagy enhances receptor for advanced glycation endproducts (RAGE)‐dependent NETosis, and pharmacological or genetic blockade of autophagy/RAGE efficiently relieves TAN‐mediated immunosuppression.^90^ Genetic studies demonstrate that myeloid‐specific autophagy deficiency impairs TAN degranulation and NADPH oxidase‑dependent ROS production, further supporting the pro‑tumour function of TAN autophagy.^91^ Collectively, these findings underscore the importance of TAN autophagy in supporting tumour immune evasion (Figure 5).

TANs are central regulators of intratumoural iron metabolism, reshaping microenvironmental iron distribution to promote tumour progression and immune suppression. TANs highly express iron transporters and serve as the primary source of intratumoural transferrin. Preliminary evidence suggests that TAN depletion reduces tumoural transferrin levels and suppresses breast cancer lung metastasis, suggesting a causal link between TAN‐dependent iron metabolism and malignant progression. Mechanistically, tumour‐secreted GM‐CSF activates JAK/STAT5β signalling to induce TAN transferrin synthesis, compensating for tumour cell iron deficiency and sustaining tumour proliferation.^92^ In addition, TAN‐derived lipocalin 2 (LCN2) sequesters extracellular iron and modulates TME immunity.^93^ Although studies have reported that LCN2 promotes tumour migration and cancer cachexia,^94^, ^95^ its specific roles in TAN‐mediated immunosuppression remain poorly defined.

Excessive iron uptake and intracellular PUFA accumulation confer intrinsic ferroptosis vulnerability to TANs, which may represent a key metabolic mechanism controlling their immunosuppressive activity.^93^ One study reported that compared with bone marrow and splenic neutrophils, intratumoural TANs significantly upregulate ferroptosis‑related genes across diverse tumour models. Although ferroptosis reduces TAN abundance, residual TANs that survive sublethal ferroptosis acquire enhanced capacity to inhibit CD8^+^ T‐cell function; ferroptosis inhibitors fully reverse this suppression and restore anti‐tumour immunity in immunocompetent mice.^96^ It can be inferred that such contradictory phenotypes are determined by microenvironmental lipid abundance, tumour oxygenation status, variable autophagic activity and tumour‑type‑specific metabolic landscapes, which collectively shape divergent ferroptosis‑mediated immune responses. In the pancreatic TME, the adipose metabolite 12,13‐dihydroxy‐9Z‐octadecenoic acid (12,13‐DiHOME) has been found to trigger PPARγ‐dependent ferritinophagy, inducing intracellular iron overload and excessive lipid peroxidation.^97^ Mechanistic studies indicate that subsequent AA‑PEox production, accompanied by elevated CXCL2, PGE_2_ and intracellular IDO levels, collectively enhances the immunosuppressive potential of ferroptosis‑experienced TANs.^76^, ^96^, ^97^ Pharmacological evidence suggested that ferroptosis inhibition efficiently reverses TAN‑dependent immune suppression and restores anti‑tumour immunity,^96^ indicating TAN ferroptosis as a promising target for metabolic immunotherapy.

Despite well‑characterised correlations between iron reprogramming, ferroptosis and TAN immune regulation (Figure 5), it should be noted that multiple critical issues remain unresolved. It remains unclear whether enhanced iron uptake actively drives pro‑tumour TAN polarisation or merely serves as a secondary adaptive response to TME stress. The heterogeneous iron metabolism and ferroptosis susceptibility across cancer types and TAN subsets lack systematic characterisation. Moreover, current evidence is insufficient regarding the functions of core ferroptosis regulators in TANs. Looking forward, beyond small‐molecule modulators, developing TAN‑specific ferroptosis‑targeted nanocarriers and reliable iron metabolic biomarkers will promote the translational advancement of TAN metabolism‐targeted immunotherapies.

### 3.6 Metabolic crosstalk between TANs and immune cells

**FIGURE 6:**
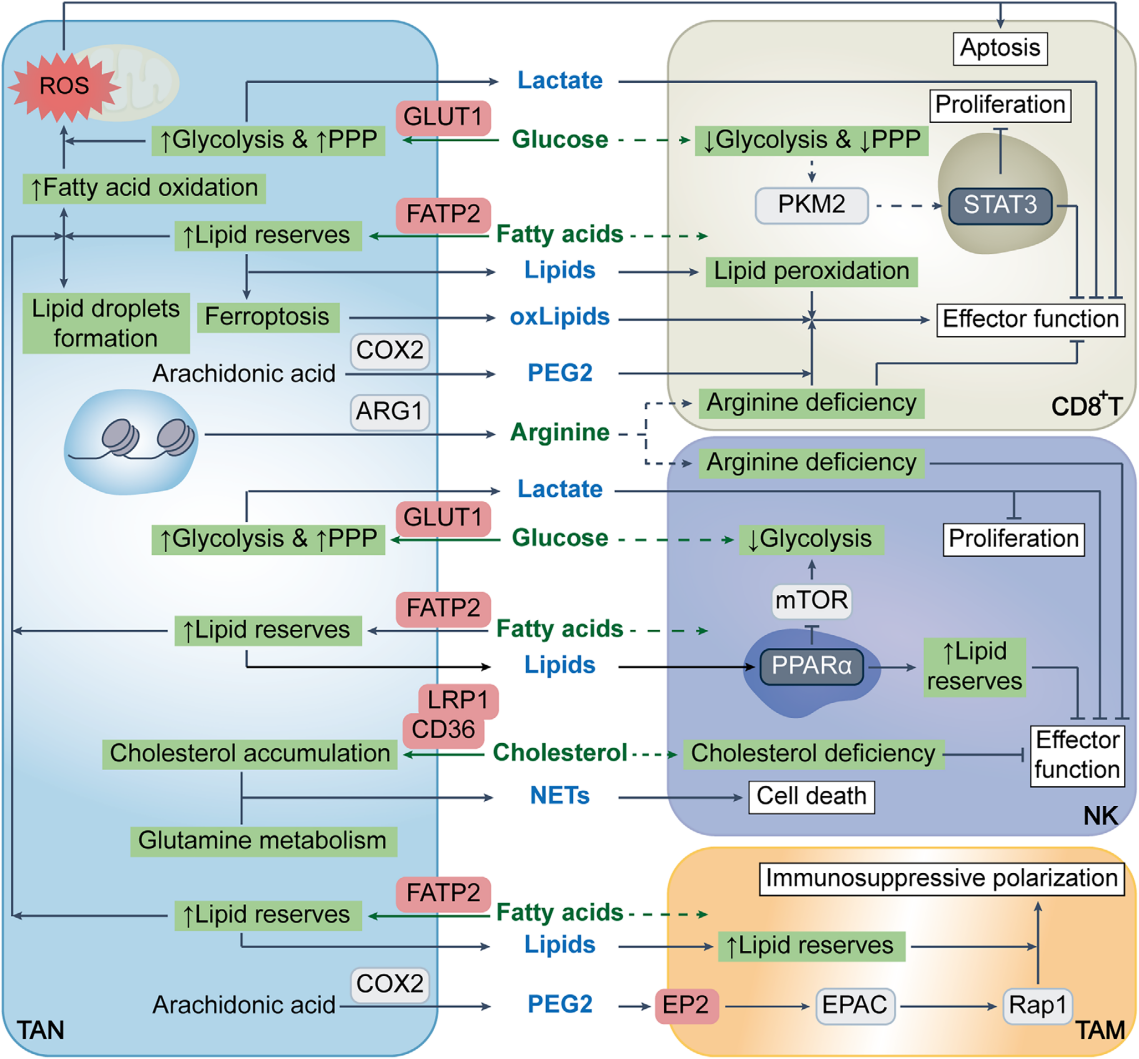
Metabolic crosstalk between TANs and intratumoural immune cells. TAN metabolic reprogramming mediates dual immunosuppressive effects via nutrient competition and metabolite‑derived signalling. TANs utilise glucose transporter 1 (GLUT1), fatty acid transport protein 2 (FATP2), cluster of differentiation 36 (CD36) and low‐density lipoprotein receptor‐related protein 1 (LRP1) to seize glucose, fatty acids and cholesterol, inducing nutrient deprivation in cytotoxic immune cells to block their proliferation and effector function. TAN‐derived lactate, prostaglandin E_2_ (PGE_2_), reactive oxygen species (ROS) and oxidised lipids further impair cytotoxic immunity. Such intercellular metabolic interplay synergistically restrains anti‐tumour immune responses and supports tumour immune escape.

The TME constitutes a sophisticated metabolic ecosystem. Beyond directly regulating immune checkpoints, TAN metabolic reprogramming triggers crosstalk with cytotoxic immune cells via nutrient competition and metabolite‑derived signalling, thereby inhibiting their effector functions and driving tumour immune evasion (Figure 6). It is generally recognised that nutrient competition is a primary driver of TAN‐mediated immunosuppression. TANs maintain hyperactive glycolysis via abundant glucose transporters and metabolic enzymes, thus outcompeting other immune cells for glucose.^98^ Glucose shortage suppresses T‐cell proliferation and induces their energy via the PKM2‐STAT5 pathway,^99^ while also impairing intratumoural NK cell proliferation.^100^ One in vitro study demonstrated that neutrophils internalise far more fatty acids than lymphocytes and accumulate correspondingly larger lipid stores.^101^ These lipid‐laden TANs transfer lipids to CD8^+^ T cells and NK cells, limiting their anti‐tumour activity, while also shuttling lipids to macrophages to drive their immunosuppressive polarisation.^78^, ^102^, ^103^ Moreover, high expression of CD36 and LDL receptor‐related protein 1 (LRP1) can lead to abnormal cholesterol buildup within TANs. Such accumulated cholesterol competes for available cholesterol and blocks lipid raft assembly and anti‐tumour signalling in NK cells; additionally, surplus cholesterol triggers NETosis, which further causes NK cell death.^104^ Immunosuppressive TANs abundantly express ARG1 to degrade extracellular arginine. Arginine starvation leads to T‐cell cycle arrest and decreased production of IFN‐γ, granzymes, and other effectors, thereby weakening the cytolytic activity of NK cells.^88^ As a pivotal iron regulator in the TME, TANs may also inhibit effector immune cells via LCN2‑mediated iron competition.^105^

TAN‐derived metabolites mediate another critical arm of metabolic crosstalk. Accumulated lactate acidifies the TME and impairs immune cell fitness.^106^, ^107^ Metabolic reprogramming, including the TCA cycle and lipid oxidation, promotes ROS production in TANs, which is suggested to induce T‐cell apoptosis and functional exhaustion.^52^, ^64^, ^78^ PGE_2_ derived from COX2‑mediated lipid metabolic reprogramming directly activates the exchange protein directly activated by cAMP (EPAC)/ras‐related protein 1 (Rap1) signalling axis in macrophages and synergises with tumour‑derived IL‐34 to drive macrophage polarisation.^108^ Moreover, TAN‑derived PGE_2_ suppresses the in vitro survival and intratumoural infiltration of CD8^+^ T cells, thereby exerting immunomodulatory functions.^65^ In parallel, various oxidised lipids released during TAN ferroptosis also act synergistically to inhibit the anti‐tumour activity of CD8^+^ T cells.^96^

It should be noted that the metabolic competition and multidimensional transcellular metabolic crosstalk within the TME are highly complex. Reprogramming of a single TAN metabolic pathway is often accompanied by compensatory effects and global metabolic network fluctuations, rather than representing an isolated, linear molecular event. This complexity constitutes a major bottleneck in dissecting the mechanistic basis of TAN metabolic heterogeneity and in developing targeted interventions. Future research should shift from descriptive phenotyping to causal mechanistic reconstruction by integrating myeloid‑specific genetic models, lineage tracing and single‑cell multi‑omics. This will enable dissection of TAN metabolic plasticity and heterogeneity, identification of specific and targetable metabolic nodes for developing TAN metabolism‐targeted immunotherapies.

## 4 SPATIOTEMPORAL HETEROGENEITY OF TAN METABOLIC REPROGRAMMING

Throughout tumour initiation and progression, TANs undergo stepwise temporal metabolic reprogramming and phenotypic transformation. Physiologically, mature circulating high‐density neutrophils (HDNs) display rigid metabolic characteristics, relying primarily on glycolysis with minimal OXPHOS, which represents the basal metabolic profile. A breast cancer study found that inflammatory factors in early TME induce the conversion of mature HDNs to iLDNs and promote bone marrow mobilisation of iLDNs. At this stage, systemic neutrophils still maintain homeostatic glycolysis‐dominant metabolism, whereas newly emerged iLDNs possess superior mitochondrial metabolism and inherent metabolic advantages over HDNs. With tumour progression, iLDNs expand dramatically and accumulate abundantly in advanced primary and metastatic lesions. Intratumoural iLDNs show concurrently elevated glycolysis and OXPHOS, increased sensitivity to glucose fluctuations, and markedly enhanced metabolic plasticity.^54^, ^109^ Another single‐cell transcriptomic study in pancreatic cancer identified three temporally distinct TAN subsets with divergent metabolic features. Early T1 subsets rely on OXPHOS, retaining the metabolic signatures of normal immature granulocytes; intermediate T2 subsets, transformed from mature neutrophils, prioritise amide and ROS metabolism to adapt to early activation after tumour infiltration; and the late‐stage T3 subset exhibits comprehensive activation of glycolysis, hypoxic stress response and pro‑angiogenic metabolic pathways.^36^ These findings suggest that TAN metabolic states exhibit context‐dependent, stage‐specific evolution during tumour progression; however, studies that directly track the in vivo temporal trajectories of TAN metabolism remain limited.

Preliminary evidence suggests that various anti‐tumour therapies can drive TAN metabolic reprogramming in a treatment‐modality‐dependent manner. Radiotherapy itself can serve as a reprogramming signal, directing TANs toward specific functional states. Subtherapeutic doses of ionising radiation drive a metabolic switch in TANs within the lung pre‐metastatic niche, marked by a shift from FAO toward glycolysis and lipid accumulation, and establish an immunosuppressive milieu that supports metastatic colonisation.^110^ Additional evidence indicates that *Glut1*‐deficient TANs enhance radiotherapy sensitivity but not anti‐PD‐1 response.^39^ Triple‐negative breast cancer cells that have acquired resistance to immunotherapy and chemotherapy accumulate neutral lipids and transmit these metabolic traits to TANs via lipid‐rich extracellular vesicles, thereby endowing them with a lipid‐laden, immunosuppressive phenotype that contributes to therapeutic resistance.^65^ However, it should be noted that the dynamic trajectories, dose‐dependent effects and molecular networks of TAN metabolic reprogramming under different therapeutic contexts remain largely unexplored.

Spatial heterogeneity is also a critical feature of TAN metabolic reprogramming. Far from being a static compartment defined by hypoxia and nutrient deprivation, the TME is dynamically divided into multiple metabolically distinct niches. Studies have shown that under chronic extreme metabolic stress with severe hypoxia and nutrient shortage, TANs infiltrating the tumour core undergo widespread metabolic rewiring characterised by hyperactive glycolysis, lactate accumulation and metabolism‐driven immunosuppression.^36^, ^48^ Spatial transcriptomic and proteomic profiling in pancreatic cancer has revealed that late‑stage T3 subsets localised in the tumour core exhibit high glycolytic activity.^36^ Another study found that in colorectal cancer, a CTCF^+^ TAN subset preferentially accumulates in the hypoxic core, where local hypoxia triggers metabolic reprogramming that confers potent pro‐oncogenic and immunosuppressive phenotypes.^111^ In contrast to the tumour core, tumour borders possess sufficient oxygen and nutrients, and border‐resident TANs adopt distinct adaptive metabolic programmes. Transitional T1 TANs at the pancreatic cancer invasive front show upregulated OXPHOS,^36^ while TANs at the gall bladder cancer border are found to exhibit aberrantly activated lipid and cholesterol metabolism.^71^ In recent years, advances in spatial metabolomics have enabled in situ characterisation of region‐specific metabolic heterogeneity. One study in the 4T1 breast cancer model, using t‐MALDI‐2 spatial metabolomics combined with single‐cell analysis, has revealed significant spatial lipid metabolic heterogeneity among TANs. Peritumoural, intratumoural active and hypoxic core regions were found to harbour distinct TAN subsets with unique lipid profiles. TAN subsets aggregated in the hypoxic core specifically upregulated the hypoxia‑associated lipid metabolite ACAR(18:0); peritumoural TAN lipid profiles were modulated by the local adipose stromal TME, while intratumoural scattered TANs exhibited relatively independent lipid metabolic features.^112^ In diabetic pancreatic cancer, spatial metabolomic studies have identified cholesterol‐enriched microdomains spatially colocalised with CXCR2^+^ TANs, where enhanced local cholesterol metabolism facilitates NETosis.^113^ Collectively, these observations suggest that the spatial heterogeneity of TAN metabolic reprogramming is not a random distribution but is closely associated with local metabolic pressures.

The establishment and maintenance of spatial metabolic features of TANs are subject to fine regulation. First, gradients of nutrient availability and cytokine concentrations guide TAN migration toward specific tumour regions, where local nutrient conditions and tumour‑derived signals may initiate adaptive metabolic reprogramming, ultimately selecting TAN subsets that optimally fit particular niches. Notably, preliminary evidence suggests that TAN glycolytic upregulation can be triggered even under normoxia in vitro, implying that acquired metabolic plasticity may precede niche adaptation rather than being a mere passive stress response, although the underlying mechanisms require further investigation.^36^ Second, studies indicate that distinct TAN subsets actively participate in constructing specific metabolic niches, thereby reinforcing spatial immunometabolic heterogeneity within TME. For instance, intratumoural TANs highly express ARG1 to deplete local L‐arginine, generating an arginine‐poor niche that paracrinely suppresses CD8^+^ T‐cell anti‐tumour activity.^86^ One study has reported that a specialised Ly6G^high^Ly6C^low^ TAN subset accumulates exclusively within intratumoural vasculature, where it senses vascular niche metabolic signals to promote NETosis; NETosis in turn triggers vascular occlusion, inducing additional intratumoural hypoxic regions and potentially influencing TAN redistribution and function.^114^

Dissecting TAN spatiotemporal metabolic heterogeneity faces prominent methodological limitations. Neutrophils’ short lifespan and low RNA content complicate sample detection, while clinical longitudinal sampling is ethically and operationally restricted. Conventional single‐cell omics only captures static cellular states, failing to track dynamic TAN metabolic shifts. Current spatial metabolomics also suffers from incomplete metabolite coverage, insufficient resolution and non‐uniform analytical standards, restricting precise identification of TAN‐specific metabolic dynamics in situ. Future improvements rely on high‐sensitivity spatial multi‐omics integrated with immunofluorescence co‐registration to match TAN cell identity and in situ metabolic phenotypes. Combined standardised pipelines, AI‐assisted analysis, intravital imaging and machine learning may overcome static detection defects, systematically decoding the molecular networks governing TAN spatiotemporal metabolic reprogramming.

## 5 METABOLISM‐BASED TAN CLASSIFICATION (4‐TIER TAN‐METAC)

Over the past decades, macrophage and T‐cell research has advanced rapidly via consensus classification systems integrating transcriptional programmes, surface markers and effector functions.^115^, ^116^ Standardised nomenclature streamlines data interpretation, cross‐study comparison and translational development to propel immunology progress. In contrast, TAN research lacks a universal unified classification framework, constituting a major bottleneck for mechanistic and translational advancement. The canonical TAN classification relies on TGF‐β‐mediated polarisation into anti‐tumour N1 and pro‐tumour N2 subsets,^117^ whose metabolic patterns are mostly deduced from phenotypic observations without direct metabolic validation. N1 TANs prioritise glycolysis and the PPP to generate metabolites supporting ROS production and proinflammatory cytokine secretion, whereas N2 TANs favour FAO to sustain long‐term survival and immunosuppression. Alternative classification schemes are also built on cell physical characteristics. HDNs exert typical anti‐tumour effector functions with steady basal metabolism, while functionally heterogeneous iLDNs generally drive immunosuppression with high metabolic plasticity.^54^ However, this classification is highly susceptible to experimental manipulation, sample processing and intratumoural hypoxia. Marker‐based subtyping also reveals discrete metabolic preferences: pancreatic P2RX1^+^ TANs display enhanced glycolysis, whereas P2RX1^−^ counterparts prefer OXPHOS,^52^ yet single‐marker typing is tumour‑type restricted and lacks cross‐platform universality. Single‐cell multi‐omics analyses further uncover widespread transcriptional heterogeneity among TAN populations (e.g., T1/T2/T3). Nevertheless, transcriptomic clustering largely reflects transient adaptive states rather than stable functional identities, and subtyping across independent studies remains inconsistent.^7^, ^36^, ^81^ Notably, the high plasticity of TANs renders the metabolic programme–functional phenotype relationship inherently context‑dependent rather than strictly unique.

**FIGURE 7:**
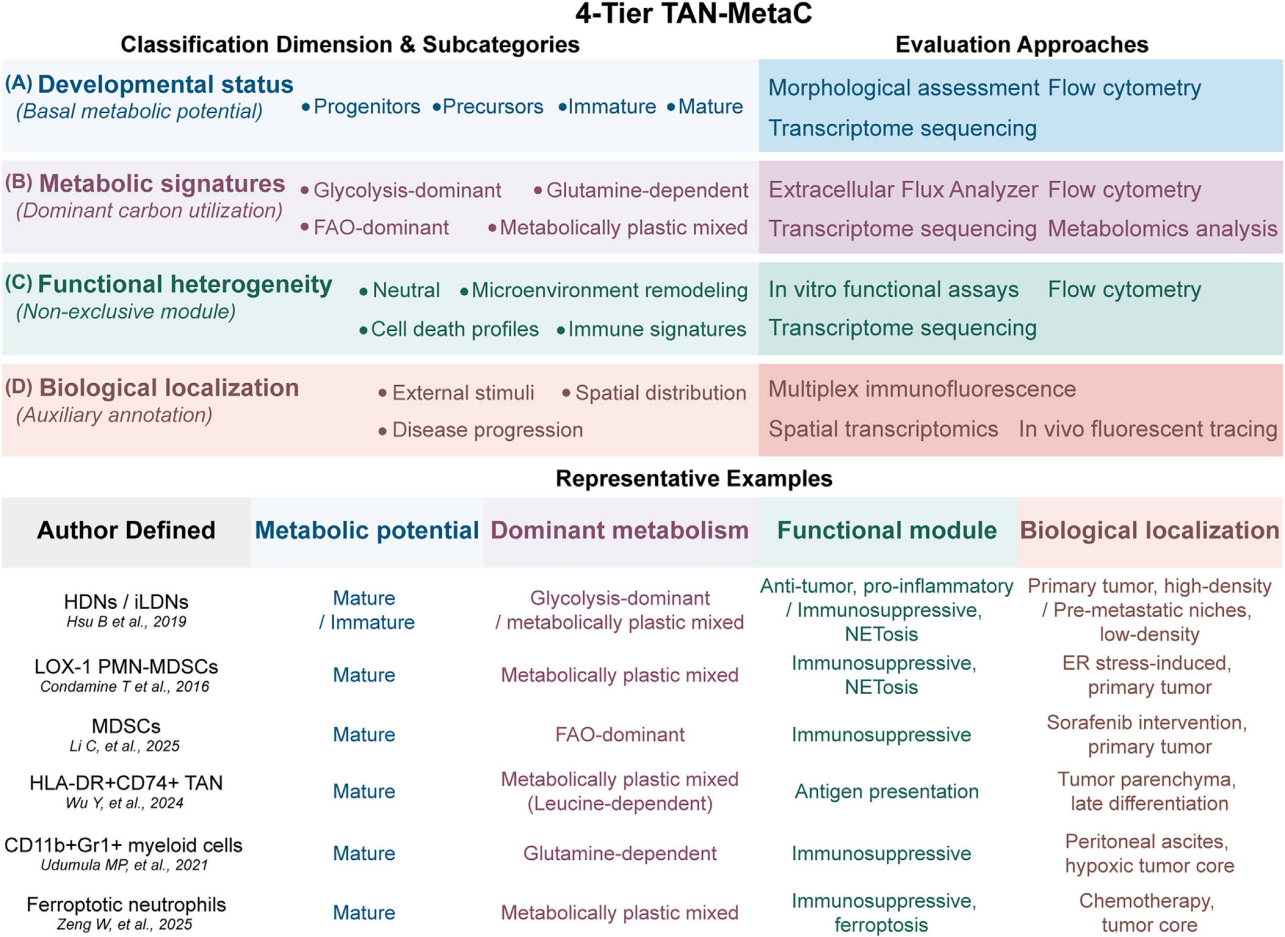
Schematic overview of the four‐tier TAN‐MetaC. The four‐dimensional system sequentially dissects TAN heterogeneity from developmental status (representing the metabolic potential), metabolic signatures (representing the dominant metabolism), functional heterogeneity (representing the functional module) to biological localisation, with dedicated experimental evaluation approaches assigned to each tier. Based on carbon substrate utilisation preference, TANs are categorised into four core metabolic subtypes: glycolysis‐dominant, fatty acid oxidation (FAO)‐dominant, glutamine‐dependent and metabolically plastic mixed subsets. A variety of previously reported neutrophil subsets are systematically annotated in this framework as representative examples. This multi‐tiered paradigm hopes to reconcile discrepancies generated by conventional single‐marker or phenotype‐dependent TAN classification, and establishes a standardised system for interpreting immunometabolic heterogeneity of TANs.

To resolve this long‐standing limitation and better capture this metabolism–function connection, we herein propose a unified metabolism‐centred hierarchical classification framework for TANs (4‐Tier TAN‐MetaC) (Figure 7). Unlike physical properties or surface markers, metabolic reprogramming is not merely a passive byproduct of cell activation, but engages in bidirectional crosstalk with signalling and transcriptional networks to collectively drive TAN polarisation.^118^, ^119^ Grounded in and reflected by relatively stable cell‐intrinsic transcriptional and functional programmes, metabolic characteristics may overcome inter‐tumoural heterogeneity and lock neutrophils into defined functional phenotypes. Indeed, multiple independent studies have established that elevated FAO represents a common metabolic feature of immunosuppressive TAN activation across diverse cancer types, further supporting the feasibility of utilising metabolic signatures as TAN classification anchors.

This four‐tier hierarchical framework dissects TAN heterogeneity sequentially by developmental status, core metabolic signatures, functional heterogeneity and biological localisation. Tier 1 (metabolic potential) serves as the foundational layer defining basal metabolic potential, classifying neutrophils into progenitors, precursors, immature and mature subsets. Developmental status shapes intrinsic metabolic preferences and eliminates developmental confounding biases in subtyping interpretation. As the core decisive layer, Tier 2 (dominant metabolism) classifies TANs into four metabolic subtypes according to dominant carbon utilisation: glycolysis‑dominant, FAO‑dominant, glutamine‐dependent and metabolically plastic mixed subtypes. Subtype assignment is defined by the core metabolic programme driving immunosuppressive and pro‐tumour functions, not by substrate availability or incidental mitochondrial flux. Glycolysis‐dominant TANs rely on glycolysis and PPP for immunosuppression; FAO‐dominant TANs show reduced glycolysis and depend on lipid droplet‐derived FAO. Glutamine‐dependent TANs sustain hyperactive TCA cycling via glutamine/glutamate. Metabolically plastic mixed TANs lack fixed addiction and represent transitional intermediate states. Built on core metabolic typing, Tier 3 (functional module) annotates functional heterogeneity via combinatorial, non‐exclusive modules covering neutral, microenvironment remodelling markers (e.g., chemotaxis, matrix remodelling, angiogenesis, hypoxic inflammation, interferon response, heat‐shock stress), cell death profiles (e.g., NETosis, ferroptosis, autophagy) and immune signatures (e.g., immunosuppression, antigen presentation). Metabolic programmes are tightly coupled with cellular functional outputs; these modules are not mutually exclusive but follow defined co‐occurrence and weak exclusion rules. Tier 4 (biological localisation) further integrates spatial distribution, disease progression and external stimuli to form a standardised multidimensional annotation system, enabling precise tracing and contextual interpretation of TAN heterogeneity across distinct biological contexts.

Despite conceptual strengths that reconcile conflicting findings from published literature, this framework still faces translational obstacles. Unified metabolic criteria and standardised detection pipelines for precise subtype assignment remain unavailable, as do convenient, clinically applicable biomarkers for subtype demarcation and therapeutic decision‐making. Overall, rooted in biological causality, this system hopes to address the persistent confusion between stable intrinsic TAN subtypes and transient microenvironment‐induced adaptive states from an immunometabolic perspective. By constructing a complete cascade connecting upstream metabolic reprogramming to intermediate functional polarisation and downstream microenvironmental adaptation, this framework delivers a robust, translationally viable theoretical basis for unifying TAN nomenclature, dissecting immunometabolic heterogeneity, and developing subtype‐targeted immunotherapies for cancers.

## 6 TARGETING TAN METABOLIC REPROGRAMMING

### 6.1 In vivo metabolic editing

Robust intratumoural TAN infiltration provides a favourable opportunity for in situ metabolic intervention.^7^, ^36^ Preclinical studies have demonstrated that systemic or local administration targeting key metabolic molecules and signalling pathways could reprogramme pro‐tumourigenic TANs into anti‐tumour phenotypes (Table 1). One study reported that the combination of TLR agonists and mitochondrial complex I inhibitors efficiently remodels TANs: TLR activation initiates NF‐κB signalling to establish cytotoxic potential, while mitochondrial complex I inhibition triggers an mROS burst, thereby eliminating tumour cells via oxidative stress storm.^120^ Preliminary evidence suggests nanobiomaterials may represent a potential strategy for precisely regulating TAN metabolism. For instance, one study demonstrated that MTP10‐HDL induces trained immunity‐mediated persistent glycolytic reprogramming and durable anti‐tumour immune memory in TANs, which is transmissible via bone marrow transplantation.^121^ Glutamine metabolism appears to be a shared and vulnerable target for both TANs and tumour cells. Both cell types rely heavily on glutamine to support energy metabolism and biosynthesis. Preclinical data indicate that in subcutaneous 4T1 breast tumour‐bearing models, the oral glutaminase antagonist JHU083 suppresses TAN recruitment and induces TAN apoptosis. In addition, JHU083 enhances T‐cell activation, and dually blocks glycolysis and oxidative metabolism in tumour cells.^122^, ^123^

**TABLE 1:** Summary of promising immunomodulatory targets of TAN metabolism.

Target	Drug	Tumour type	Mechanisms	Ref
FATP2	Tumour‐derived exosomal circRNA_0013936	Bladder cancer	Inhibiting the synthesis of immunosuppressive molecules (ARG1, IL‐10, iNOS) of TANs	PMID: 38461272
	Lipofermata	Melanoma; lung cancer	Inhibiting lipid accumulation and ROS formation, blocking immunosuppressive activity of TANs Enhancing αPD‐L1 tumour immunotherapy via the upregulation of CD107a and reduced PD‐L1 expression on CD8^+^ T cells	PMID: 33524739
	Lipofermata	Lymphoma; lung cancer; colon cancer; pancreatic cancer	Regulating uptake of arachidonic acid and PGE_2_ synthesis of TANs Enhancing αCTLA4 tumour immunotherapy via regulating the infiltration and function of CD8^+^ T cells	PMID: 30996346
ARG1	hARG1‐specific monoclonal antibody (mAb)	Pancreatic cancer	Inhibiting the enzymatic activity of ARG1 released by TANs Promoting the frequency of adoptively transferred tumour‐specific CD8^+^ T cells in tumour Enhancing the effectiveness of immune checkpoint therapy	PMID: 36921034
	CB‐1158	Pan‐cancer	Inhibiting the enzymatic activity of ARG1 released by TANs Increasing tumour‐infiltrating CD8^+^ T cells and NK cells, inflammatory cytokines, and expression of interferon‐inducible genes	PMID: 29254508
	Neutrophil‐activating therapy	Melanoma	Inducing ROS generation of TANs	PMID: 36706760
NAMPT	FK866	Melanoma	Inhibiting SIRT1‐mediated transcription of proangiogenic genes	PMID: 30121947
TGF‐β1 receptor	dMSN‐SB	Pancreatic cancer	Inhibiting ARG1 expression of TANs Enhancing tumour response to combined IRE and αPD1 therapy Inducing long‐term anti‐tumour memory	PMID: 35128843
HDAC	CN133	Prostate cancer	Inhibiting ARG1 expression of TANs Enhancing αPD1 efficacy in subcutaneous prostate cancer allografts	PMID: 37880708
STAT5	GTN	Breast cancer	Inducing ROS generation of TANs Modulating FATP2 expression of TANs Enhancing the immune‐mediated anticancer therapy	PMID: 37370739
Ca5	Cyclic hexapeptide AcF(OP(D)ChaWR)	Lung cancer	Modulating immunomodulators ARG1, CTLA‐4, IL‐6, IL‐10, LAG3 and B7H1 expressions	PMID: 23028051
ACOD1	Gene knockout	Breast cancer	Activating Nrf2‐mediated antioxidant response	PMID: 37793345
ACKRs	Gene knockout	Breast cancer	Inducing ROS production	PMID: 29445158

Abbreviations: ACKRs, atypical chemokine receptors; ACOD1, aconitate decarboxylase 1; ARG1, arginase 1; Ca5, carbonic anhydrase 5; FATP2, fatty acid transport protein 2; hARG1, human arginase 1; HDAC, histone deacetylase; iNOS, inducible nitric oxide synthase; IRE, irreversible electroporation; NAMPT, nicotinamide phosphoribosyltransferase; PD‐L1, programmed death‐ligand 1; ROS, reactive oxygen species; SIRT1, sirtuin 1; STAT5, signal transducer and activator of transcription 5; TGF‐β1, transforming growth factor‐beta 1; αCTLA4, anti‐cytotoxic T‐lymphocyte‑associated protein 4 antibody; αPD1, anti‐programmed cell death protein 1 antibody.

Despite promising preclinical efficacy, multiple obstacles hinder clinical translation. First, the lack of TAN‐specific therapeutic targets induces nonspecific pleiotropic effects throughout the TME. Meanwhile, current delivery systems fail to achieve precise TAN targeting, frequently resulting in off‐target drug accumulation and systemic toxicity. Second, the TAN metabolic network exhibits high plasticity, potentially allowing cells to swiftly engage compensatory pathways when a single metabolic node is inhibited, thus undermining the therapeutic effect. Third, the short lifespan of TANs prevents stable function after transient metabolic intervention, resulting in rapid efficacy attenuation. Future research is required to develop TAN‐specific delivery vehicles (e.g., lipid nanoparticles conjugated with Ly6G/CD11b antibodies^124^) and explore strategies to reinforce metabolic memory (such as combination with histone deacetylase [HDAC] inhibitors to stabilise epigenetic modifications), so as to lock the anti‐tumour phenotype within the limited lifespan of TANs.

### 6.2 Engineered cellular therapies

Engineered cellular therapy remodels the metabolism and function of neutrophils or their progenitor cells in vitro before adoptive transfer. Current engineering strategies are mainly divided into two approaches. In the first approach, bone marrow progenitor cells are trained in donor animals via exogenous stimuli or endogenous genetic editing, and mature TANs are subsequently isolated for adoptive transfer. One study reported that β‐glucan pretreatment remodels the transcription and epigenetics of bone marrow progenitors, driving neutrophil differentiation into ROS‐dependent anti‑tumour subtypes with transmissible trained immunity via bone marrow transplantation.^125^ Another study found that adoptive transfer of *Acod1*‐deficient neutrophils suppresses lung metastasis and prolongs host survival, while wild‐type TANs exert an opposite pro‐metastatic effect, providing preclinical evidence for TAN mitochondrial metabolism‐based cell therapy.^55^ In the second approach, preliminary evidence suggests that human pluripotent stem cells (hPSCs) are genetically edited and directionally differentiated into functional neutrophils, among which CAR‐neutrophils emerge as a cutting‐edge direction.^126^ The use of hPSCs has addressed the challenge of obtaining a stable and renewable cell source. Moreover, CAR‐neutrophils possess superior capacities, including robust infiltration into hypoxic solid tumour regions, low treatment‐related toxicity and tumour‐targeted drug delivery. Mechanistically, hPSC‐derived CAR‐neutrophils couple CAR‐triggered spleen tyrosine kinase (SYK)/extracellular regulated‐protein kinases (ERK) signalling with intracellular metabolic networks, sustaining stable ROS production under hypoxia. These cells also accurately home to tumour lesions, penetrate biological barriers and release tumouricidal mediators.^127^

Despite the promising outlook of these strategies, their translational maturity requires rigorous evaluation at the early preclinical stage. First, critical technical bottlenecks remain regarding cell sources and manufacturing protocols, among which the scalability of hPSC‐derived neutrophil production (including differentiation efficiency, batch‐to‐batch consistency, and cost control) remains a major barrier to large‐scale clinical application. Second, therapeutic efficacy fades rapidly following adoptive transfer, and the high plasticity of neutrophils renders them susceptible to microenvironmental cues, potentially driving secondary phenotypic shifts. Without sustained in vivo supportive cues or repeated infusions, a single adoptive transfer may only afford a narrow therapeutic window. Notably, a recently developed CLON‐G inhibitor cocktail (consisting of caspase inhibitors, LMP inhibitors, oxidative necroptosis inhibitors and G‐CSF) has succeeded in extending the in vitro lifespan of neutrophils to 5–7 days while preserving their chemotactic capacity and ROS production, laying actionable technical foundations for adoptive transfer regimens.^128^ Third, the metabolic cost imposed by constitutive CAR signalling cannot be overlooked.^129^ While constitutive CAR signalling may boost glycolytic flux, leading to intracellular buildup of acidic metabolites that drive neutrophil apoptosis and aberrant NETosis, thereby imposing risks of metabolic toxicity.

### 6.3 Genetic validation of target specificity: Evidence from knockout models

Compared with pharmacological intervention, CRISPR‐Cas9‐based cellular models and tissue‐specific knockout (KO) animal models serve as an important standard for distinguishing cell‐intrinsic metabolic effects from indirect systemic alterations. Neutrophil‐specific KO models effectively circumvent the off‐target liabilities of small molecule inhibitors and enable unambiguous assignment of metabolic gene functions to TANs. As summarised in Table 2, multiple metabolic targets have been functionally validated through global, myeloid‐restricted, or neutrophil‐specific ablation strategies. Global ablation of *Hif1a* causes lethal vascular leakage, whereas myeloid‐specific *Hif1a* knockout safely reverses the pro‐tumour glycolytic signature of TANs and modulates ROS release as well as the effector functions of immunotoxic cells.^46^ FATP2 is highly enriched in TANs. Myeloid‐specific *Slc27a2* deletion effectively abrogates TAN‐mediated suppression of CD8^+^ T‐cell proliferation, while leaving macrophage functions intact, suggesting a TAN‐restricted functional dependency. This effect is target‐specific, as deletion of alternative lipid transporters FATP4 or CD36 fails to recapitulate these anti‐tumour functional phenotypes.^61^ Notably, preclinical models have uncovered latent risks associated with single metabolic gene knockout. For instance, neutrophil‐specific deletion of *Acod1* augments systemic proinflammatory cytokine secretion and exacerbates multi‐organ injury under inflammatory conditions. Depletion of *Acod1*‐derived itaconate abolishes the intrinsic inhibitory machinery of NETosis; meanwhile, loss of Acod1 elevates peptidylarginine deiminase 4 abundance, ultimately driving excessive NETosis.^130^ Therefore, ablation of a single metabolic gene may disrupt the endogenous protective programmes and trigger uncontrolled toxic phenotypes. Careful target screening and rigorous risk assessment are therefore indispensable for future translational applications.

**TABLE 2:** Summary of genetic knockout models for validating TAN‐specific target function.

Model type	Target gene	Cre driver/editing tool	Cell population affected	Key conclusion on target specificity	Ref
Neutrophil‐specific conditional KO	Hif1a	Mrp8‐Cre	Only neutrophils	Partial deletion (∼50% knockdown) of *Hif1a* specifically in pancreatic tumour‐associated neutrophils	PMID: 36614196
Global whole‐body KO	Ppara	CRISPR‐Cas9	All somatic cells	Pparα is preferentially expressed in neutrophils; Pparα^high^ neutrophils exhibit enhanced immunosuppressive capacity. Mixed bone marrow chimeras demonstrated that Pparα^−^^/^^−^ neutrophils were less immunosuppressive than their WT counterparts. Pharmacological inhibition with GW6471 recapitulated the loss of neutrophil function, while neutrophil depletion attenuated GW6471‐induced tumour suppression	PMID: 39876004
Global whole‐body KO	P2rx1	CRISPR‐Cas9	All somatic cells	P2rx1 showed the highest expression intensity in neutrophils relative to other immune cell populations. Bone marrow chimera experiments revealed that loss of *P2rx1* in haematopoietic cells sufficed to drive metastasis. Neutrophil depletion eliminated the differential metastatic phenotypes between WT and P2rx1^−^^/^^−^ mice	PMID: 33420030
Global whole‐body KO/Myeloid conditional KO/Neutrophil‐specific conditional KO	Acod1	CRISPR‐Cas9/LysM‐Cre/Mrp8‐Cre	All somatic cells/All myeloid cells/Only neutrophils	Adoptive transfer of tumour‐primed Acod1^−^^/^^−^ BMDNs into either WT or Acod1^−^^/^^−^ recipients consistently reduced lung metastasis. Mrp8‐Cre^+^Acod1^fl/fl^ mice fully recapitulated the global knockout phenotype. Both Mrp8‐Cre and LysM‐Cre produced equivalent phenotypes, excluding monocyte/macrophage contribution	PMID: 37793345
Neutrophil‐specific conditional KO	Slc27a2 (FATP2)	S100A8‐Cre	Only neutrophils	Fatp2 expression is selectively enriched in neutrophils. *Fatp2* deletion selectively eliminates neutrophil suppressive function. Neither *Fatp4* nor *Cd36* deficiency phenocopied Fatp2 ablation, establishing Fatp2 as a non‐redundant target in neutrophil‐mediated immunosuppression	PMID: 30996346
Neutrophil‐specific conditional KO	Slc2a1 (Glut1)	Ly6G‐Cre	Only neutrophils	Ly6G‐Cre‐mediated *Glut1* deletion specifically abrogated the pro‐survival effect of tumour supernatant on neutrophils. G*lut1*‐deficient neutrophils showed a significant reduction in the SiglecF^high^ subset and accelerated turnover in the TME, identifying Glut1 as the predominant glucose transporter in long‐lived neutrophils	PMID: 33753374
Pan‐haematopoietic conditional KO/Neutrophil progenitor‐specific KO/Inducible haematopoietic KO/In vitro single‐cell KO	Atg7/Atg5	Vav‐Cre/Cebpa‐Cre/Mx1‐Cre/CRISPR‐Cas9	All haematopoietic stem and progenitor cells/GMP and downstream neutrophil progenitors/Inducible haematopoietic cells/32D myeloblast cell line	Cebpa‐Cre (neutrophil progenitor‐restricted) and Vav‐Cre (pan‐haematopoietic) both caused consistent differentiation arrest. Inducible *Atg5* deletion and CRISPR‐edited 32D cells recapitulated the defect in vitro and in vivo. Cebpa‐Cre targets the GMP stage rather than HSCs, collectively demonstrating that the phenotype is cell‐intrinsic and independent of HSC autophagy	PMID: 28916263
Neutrophil‐specific conditional KO	Junb	Mrp8‐Cre	Only neutrophils	Adoptive transfer of *Junb*‐deficient neutrophils into WT recipients failed to upregulate immunosuppressive and angiogenic gene signatures. Mrp8‐Cre^+^Junb^fl/fl^ mice recapitulated the functional loss observed in global perturbation models. Junb ablation did not impair neutrophil differentiation but selectively eliminated their immunosuppressive and pro‐angiogenic activities	PMID: 41339555
Global whole‐body KO	Ager	ES cell‐based homologous recombination	All somatic cells	Rage^−^^/^^−^ neutrophils displayed impaired NETosis. RAGE loss led to decreased serum DNA and CitH3 levels in vivo. However, given the global knockout strategy, the contribution of Rage from non‐neutrophil cell types cannot be fully excluded; neutrophil‐specific conditional knockout is warranted to conclusively establish cell‐intrinsic specificity	PMID: 25908451
Global whole‐body KO	Ackr2	ES cell‐based homologous recombination	All somatic cells	Bone marrow chimera experiments revealed that haematopoietic *Ackr2* deletion alone sufficed to confer metastasis protection. Neutrophil depletion reversed the protection in Ackr2^−^^/^^−^ mice, while monocyte or B‐cell depletion did not. Adoptive transfer of Ackr2^−^/^−^ neutrophils, but not WT neutrophils, reduced metastasis in WT recipients	PMID: 29445158

Abbreviations: BMDNs, bone marrow‑derived neutrophils; CitH3, citrullinated histone H3; Cre, Cre recombinase; CRISPR‑Cas9, clustered regularly interspaced short palindromic repeats/CRISPR‑associated protein 9; ES cell, embryonic stem cell; fl/fl, floxed (loxP‑flanked); GMP, granulocyte‑monocyte progenitor; HSCs, haematopoietic stem cells; i.p., intraperitoneal; KO, knockout; NETosis, neutrophil extracellular traps; TME, tumour microenvironment; WT, wild‑type.

### 6.4 Translational metabolic engineering: Future perspectives

**FIGURE 8:**
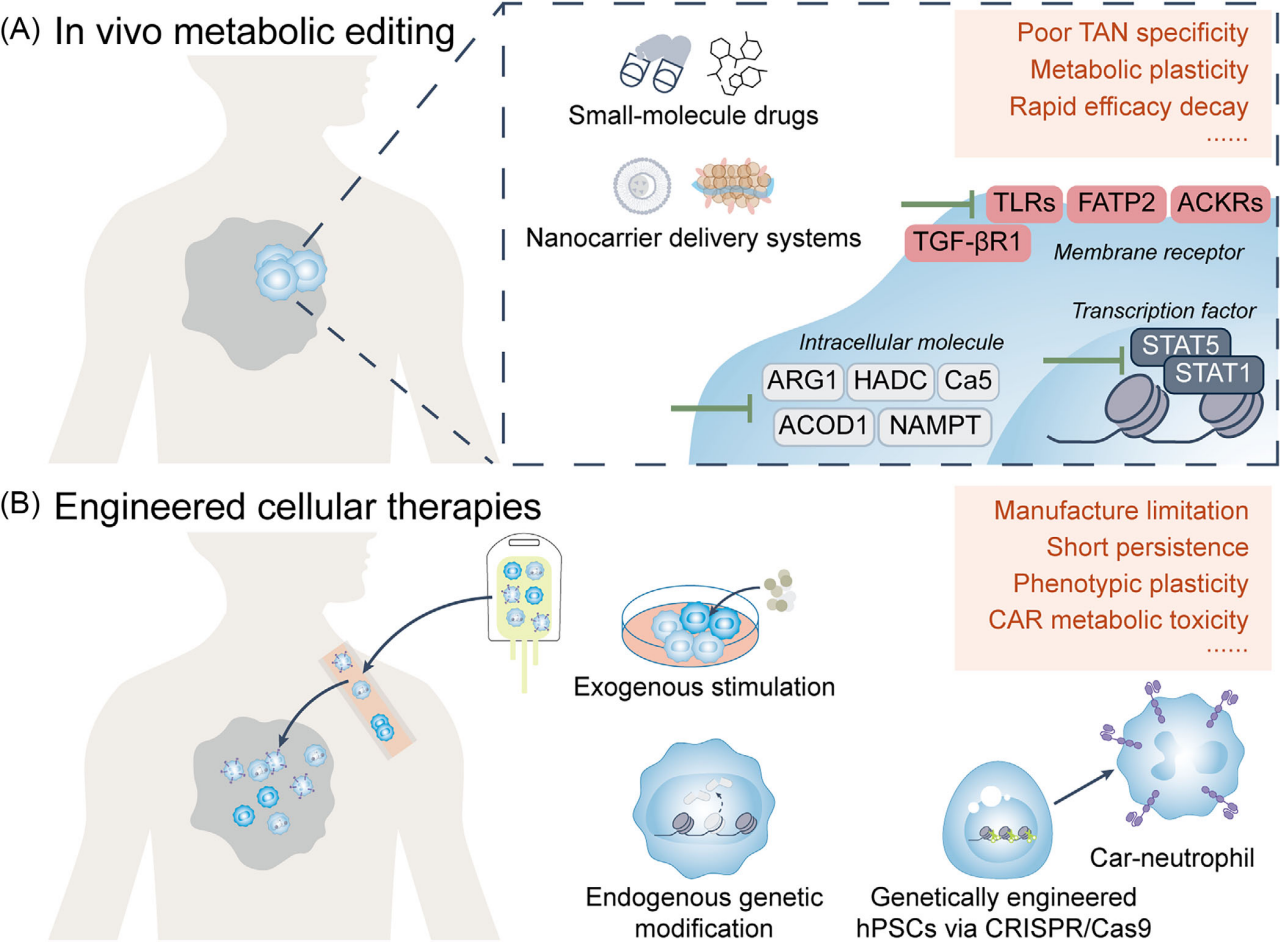
TAN metabolism‐targeted immunotherapies. (A) In vivo metabolic editing. Small‐molecule drugs or nanocarriers intervene tumour‐associated neutrophil (TAN) metabolism by targeting receptors (e.g., fatty acid transport protein 2 [FATP2]), intracellular molecules (e.g., arginase 1 [ARG1]) and transcription factors (e.g., signal transducer and activator of transcription 5 [STAT5]); translational barriers include insufficient TAN‑specific targeting, metabolic plasticity and rapid efficacy decay. (B) Engineered cellular therapies. Functional therapeutic neutrophils are obtained via endogenous genetic modification, exogenous stimulation or chimeric antigen receptor (CAR)‐neutrophil generation from gene‐edited human pluripotent stem cells (hPSCs), facing hurdles of manufacture limitation, short cell persistence, phenotypic plasticity and CAR metabolic toxicity.

Although encouraging advances have been achieved in preclinical research (Figure 8) and several relevant clinical trials are underway (Table 3), TAN‐directed metabolic interventions still confront fundamental translational hurdles, including the short in vivo lifespan of neutrophils, insufficient TAN‑specific targeting, unstable anti‐tumour phenotypes and limited capacity for ex vivo cellular expansion. We propose several top‐priority research directions for future work: First, construct a unified TAN subtyping framework integrated with multi‐omics, functional metabolic assays and spatial analysis. This standardisation will enable cross‐study comparability and facilitate subset‐specific precise therapeutic targeting. Second, develop in situ metabolic imaging platforms and neutrophil‑exclusive delivery systems exemplified by Ly6G/CD11b antibody‑conjugated lipid nanoparticles. Third, integrate dual‐dimensional neutrophil metabolic engineering strategies to simultaneously lock stable anti‐tumour phenotypes and extend functional lifespan. On one front, metabolic preconditioning during ex vivo culture, such as blocking LDHA, ATP‐citrate lyase or other key metabolic nodes, may suppress histone lactylation and acetyl‐CoA metabolic dysregulation, thereby pre‐adapting TANs to the TME and preventing their pro‐tumourigenic polarisation. On another front, targeting the PI3Kγ/AKT/mTOR axis while reinforcing FAO can prolong the functional persistence of TANs, thereby widening the therapeutic window. Concurrently, adjunctive measures, including the depletion of cytokines that drive pro‐tumour polarisation or the application of CLON‐G inhibitor cocktails, may consolidate the efficacy of CAR‐neutrophil therapy. In parallel, rigorous evaluations of pharmacotoxicity and immunogenicity should be carried out in humanised mouse models to inform clinical translation. Collective advances across all these dimensions are indispensable to translate fundamental TAN immunometabolic discoveries into safe, clinically viable therapeutic regimens.

**TABLE 3:** Summary of ongoing or completed clinical trials targeting TAN metabolism.

Target	Drug	Tumour type	Trial identifier	Phase	Study status
ARG1	INCB001158/CB‐1158	Advanced/metastatic solid tumours	NCT02903914	Phase I	Completed
	INCB001158 + chemotherapy	BTC, CRC, ovarian, gastroesophageal, endometrial cancer and other solid tumours	NCT03314935	Phase I/II	Completed
	INCB001158 + epacadostat ± pembrolizumab	Advanced/metastatic solid tumours	NCT03361228	Phase I/II	Terminated
	INCB001158 + retifanlimab	Advanced/metastatic solid tumours	NCT03910530	Phase I	Completed
PI3Kγ	IPI‐549/eganelisib + nivolumab	Advanced solid tumours; high‐circulating MDSC cohort	NCT02637531	Phase I	Unknown
	IPI‐549/eganelisib + atezolizumab ± nab‐paclitaxel/bevacizumab	TNBC; RCC	NCT03961698	Phase II	Unknown
	IPI‐549/eganelisib + nivolumab	Advanced urothelial carcinoma	NCT03980041	Phase II	Completed
TGF‐β/TGF‐βR	Galunisertib/Vactosertib	PC, NSCLC, GC and other solid tumours	NCT02734160; NCT02304419; NCT04515979; NCT04893252	Phase I; Phase I; Phase II; Phase II	Completed; Completed; Terminated; Unknown
	Bintrafusp alfa/M7824	CRC/MSI‐H solid tumours; mesothelioma; NSCLC and other solid tumours	NCT03436563; NCT05005429	Phase I/II; Phase II	Completed
	NIS793/SAR439459/AVID200/Fresolimumab	Advanced malignancies, advanced solid tumours, BC, early‐stage NSCLC	NCT02947165; NCT03192345; NCT03834662; NCT01401062; NCT02581787	Phase I; Phase I; Phase I; Phase II; Phase I/II	Completed
HDAC	Entinostat + nivolumab/ipilimumab	Metastatic/unresectable solid tumours; HER2‐negative BC	NCT02453620	Phase I	Active, not recruiting
A2A receptor	Ciforadenant ± atezolizumab	RCC; metastatic castration‐resistant PC	NCT02655822	Phase I/Ib	Completed

Abbreviations: A2AR, adenosine A2A receptor; ARG1, arginase 1; BC, breast cancer; BTC, biliary tract cancer; CRC, colorectal cancer; GC, gastric cancer; HDAC, histone deacetylase; MDSC, myeloid‐derived suppressor cell; MSI‐H, microsatellite instability‐high; NSCLC, non‐small cell lung cancer; PC, pancreatic cancer; PD‐1, programmed cell death protein 1; PD‐L1, programmed death‐ligand 1; PI3Kγ, phosphoinositide 3‐kinase gamma; RCC, renal cell carcinoma; TGF‐β, transforming growth factor‐beta; TGF‐βR, transforming growth factor‐beta receptor; TNBC, triple‐negative breast cancer.

## 7 CONCLUSION

TAN metabolic reprogramming represents an emerging frontier in cancer immunometabolism. The metabolic features of TANs are characterised by high plasticity and spatiotemporal heterogeneity. This metabolic reprogramming, whether actively driven or passively acquired, governs the functional fate of TANs in tumour immunity and contributes to sculpting the overall immune landscape through multiple pathways. Recent advances, spanning single‐cell metabolomics and spatial imaging, have substantially expanded our understanding of the intricate molecular networks underlying TAN metabolic remodelling and its intimate relationship with immunosuppressive functions. These insights have laid a solid theoretical foundation for targeted therapeutic development.

Despite considerable promise from preclinical data, the field remains nascent, and the translational path warrants rigorous and systematic validation, including the substantial heterogeneity of TANs, the lack of effective monitoring and tracking tools, insufficient TAN‐specific targeting, and the short lifespan of neutrophils. To move toward actionable investigation, we propose several concrete research priorities: (i) establish a unified TAN subtyping framework; (ii) develop in situ metabolic imaging platforms, such as spatial metabolomics and bioluminescent probes; (iii) design lineage‐tracing studies coupled with metabolic labelling to dynamically track the ontogeny and metabolic trajectories of TAN subsets throughout tumour progression and therapeutic intervention; (iv) develop precision targeting technologies to improve delivery specificity; and (v) implement metabolic engineering strategies, including extending neutrophil lifespan and stabilising immunosuppressive phenotypes. Looking ahead, precisely targeting TAN metabolic vulnerabilities through these coordinated efforts holds promise for developing next‐generation immunotherapeutic strategies to improve cancer patient outcomes.

## AUTHOR CONTRIBUTIONS

**Wenchao Xu**: conceptualisation, investigation, writing—original draft, funding acquisition. **Yu Su**: writing—review and editing. **Li Zhou**: investigation, validation. **Jianzhou Liu**: writing—review and editing. **Junchao Guo**: supervision, writing—review and editing, project administration, funding acquisition.

## CONFLICT OF INTEREST STATEMENT

The authors declare no conflicts of interest.

## ETHICS STATEMENT

Ethical approval is not applicable to this review article.

## Data Availability

Data sharing does not apply to this article as no new datasets were generated or analysed during the current study.
